# Effect of Chalcogenophenes on Chiroptical Activity of Twisted Tetracenes: Computational Analysis, Synthesis and Crystal Structure Thereof

**DOI:** 10.3390/molecules28135074

**Published:** 2023-06-28

**Authors:** Gayathri Jothish Kumar, Benny Bogoslavsky, Sashi Debnath, Anjan Bedi

**Affiliations:** 1 Department of Chemistry, SRM Institute of Science and Technology, Kattankulathur, Chennai 603203, India; 2 Institute of Chemistry, The Hebrew University of Jerusalem, Edmond J. Safra Campus, Jerusalem 91904, Israel; 3 Department of Radiology, University of Texas Southwestern Medical Center, Dallas, TX 75390, USA

**Keywords:** tetracene, chalcogenophene, Suzuki coupling, twisted acenes, chiroptical properties

## Abstract

The synthesis of multiply substituted acenes is still a relevant research problem, considering their applications and future potential. Here we present an elegant synthetic protocol to afford tetra-peri-substituted naphthalene and tetracene from their tetrahalo derivatives by a Pd(0)-catalyzed C-C cross-coupling method in a single step. The newly synthesized tetracenes were characterized by NMR, HRMS, UV-vis spectrophotometry, and single-crystal X-ray diffraction (SCXRD). In addition, the first systematic computational study of the effect of chalcogenophenyl substitutions on the chiroptical properties of twistacenes was reported here. The gas phase computational studies using density functional theory (DFT) on a series of chalcogenophene-substituted tetracenes revealed that their chiroptical activity could be systematically increased via the atomistic tuning of peripheral substituents.

## 1. Introduction

Polycyclic aromatic hydrocarbons (PAHs) in the field of organic electronics are considered the Holy Grail because of their tunable optoelectronic properties resulting from their unique structure, mechanical flexibility, solution processability, etc. [1,2]. The widespread applications of PAHs include organic thin film transistors (OTFTs) [3], organic light-emitting diodes (OLEDs) [4], perovskite solar cells [5], third-generation photovoltaics or singlet-fission materials [6], TADF emitters, etc. [7]. While the exquisite properties of the acenes are anticipated for their flat, rigid π-conjugated backbones with reasonably low HOMO-LUMO gaps, the discussion on the possible existence of a core-twisted acene backbone in the solid state (arising from the steric factor in multi-peri-substituted acenes) is often understated [8]. Pascal et al. explored the structural aspects of twisted acenes (twistacenes) extensively, in which the highly substituted acene core exhibited deviation from its planarity [9]. In multi-peri-substituted acenes, twisting of the core could result in appreciable tuning in the optoelectronic and chiroptical properties [10,11,12,13]. Twisting a PAH out of planarity adds up newer properties such as increasing triplet yields by increasing inter-system crossing (ISC) [14], localizing electron density at the central benzene ring [15], and increasing the Cotton effect and dissymmetry factors in cases of both absorption (*g*_abs_) [16] and emission (*g*_lum_). These properties could propel the twistacenes into applications such as circularly polarized light emitting diodes (CPOLEDs) [17], circularly polarized phosphorescent light emitting diodes (CPPHOLEDs) [18], organic field effect transistors (OFETs) [19], thermally activated delayed fluorescence (TADF) emitters [20], materials with chirality-induced spin selectivity (CISS) effect [21,22], Faraday rotators [23], photodynamic therapy [24], etc. Recently, we unraveled that the chiroptical activity in twisted acenes could be modulated by (i) longitudinally twisting an acene core [11,16], (ii) varying substitutions around the core, and (iii) increasing the chain length of the twisted acene oligomers [25]. Furthermore, the interaction of circularly polarized light with a chirally modified conductive surface was modeled by the adsorption of different enantiomers of helically locked, tethered, twisted anthracenes [26]. Such systematic studies on longitudinally twisting an acene are limited to anthracenes, anticipating synthetic difficulties to achieve helically locked higher acenes. On the other hand, despite a large amount of recent research on tuning the optoelectronic properties by substitution on the acene [27,28], there are no reports on modulating its chiroptical activity by changing substitutions around the core in higher acenes in an atomistic manner. This is due to associated limitations in accessing helically locked chiral acenes higher than anthracene.

For atomistic tuning of π-conjugated molecules or polymers, modulating the π-delocalization via changing heteroatoms in chalcogenophenes has been successfully strategized in several studies to result in moderate to high alterations in structural, optoelectronic, and electronic device properties [29,30,31,32,33]. The fact that the effect of chalcogenophene substitution on the chiroptical properties of the acenes has not been reported to date may be due to the associated challenges in synthesis. A general method to synthesize highly substituted twistacenes involves Diels-Alder reactions of pre-modified and sterically crowded PAH synthons followed by an aromatization step [11]. However, the post-modification of the acene core is non-trivial, forcing the substitutions to be installed on it before or during the aromatization step [34]. On the other hand, the effort of the addition of multiple aryl substituents to multiply halogen-substituted acenes could lead to asymmetric products via the penta-annulation pathway [35]. Douglas et al. reported the synthesis of indeno-annulenes through a chemoselective Kumada-Tamao-Corriu coupling. In this case, the C-H activation cascade reaction has one of the significant drawbacks of uncontrolled tetra-substitution on the parent acene. In this context, Mamada et al. reported the synthetic route to attach four chalcogenophene rings at the peri-positions of tetracenes in a single step and proved that those tetracenes are photochemically more stable than rubrene [36]. However, the possibility of expanding this method to various substrates has not been reported. Additionally, there is scope for improving the method by replacing the toxic stannyl derivatives [37] and pyrophoric tri-*^t^*butylphosphines. Therefore, we present a unique protocol to achieve tetra-peri-substituted tetracenes in a single step under variable high-yield substrates and study their solid-state morphology. Furthermore, the effect of the change in heteroatoms in the heteroaryl substitutions on the chiroptical properties has been studied computationally.

## 2. Results

**Scheme 1:**
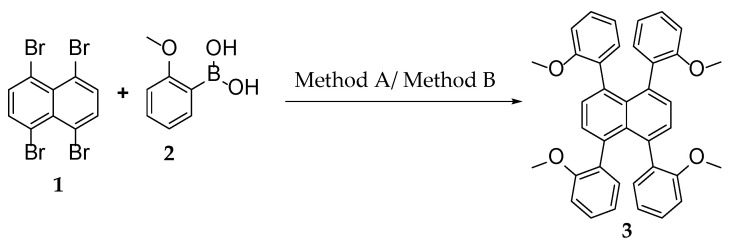
Synthesis of **3**. Method A: Pd (PPh_3_)_4_, Na_2_CO_3_, 1,4-dioxane: water (1:1), Ar, 90 °C, 30 h, 22%. Method B: Pd_2_(dba)_3_, XPhos, Na_2_CO_3_, toluene/THF/water (3:2:1), Ar, 95 °C, 13 h, 72%.

**Scheme 2:**
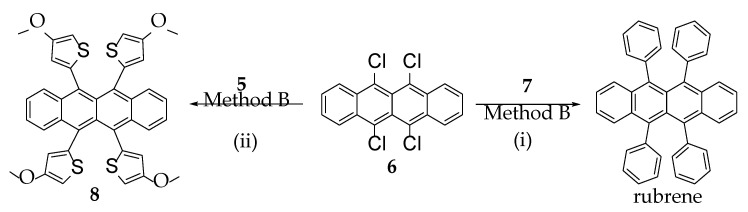
Synthesis of rubrene and **8**. Conditions: (i) phenylboronic acid (**7**), Pd_2_(dba)_3_, X-Phos, Na_2_CO_3_, toluene/THF/water (3:2:1), Ar, 95 °C, 13 h, 71%; (ii) 4-methoxythiophene-2-boronic acid (**5**) Pd_2_(dba)_3_, X-Phos, Na_2_CO_3_, toluene/THF/water (3:2:1), Ar, 95 °C, 13 h, 71%.

Recently, helically locked core twisted anthracenes with variable substituents on the acene core, synthesized by us, involved severe synthetic challenges that resulted in low to decent overall yields [11]. A twist could be enforced upon the acene core only if there are at least two bulky substituents installed on the consecutive peri positions to sterically repel each other [38]. Higher the substitutions, higher is the core twist, and lower could be the yield of their synthesis. A previous report on attempting selective tetra-peri substitutions on 1,4,5,8-tetrabromonaphthalene via Suzuki coupling by Cui et al. resulted in peri-annulation [39]. Keeping these difficulties in mind, we started the synthesis of tetraaryl-substituted naphthalene from its tetrabromo counterpart by a modified Suzuki-Miyaura coupling method. When 1,4,5,8-tetrabromonaphthalene [40] was treated with *o*-anisyl boronic acid in the presence of tetrakis(triphenylphosphine)palladium(0) as a catalyst, dioxane/water as the solvent, and Na_2_CO_3_ as the base, it afforded compound **3** with a 22% yield (Scheme 1), which was confirmed by NMR, MS, and single crystal X-ray diffraction (SCXRD). The identical approach to synthesizing rubrene from tetrachlorotetracene [41,42] failed, leading to a mixture of unassigned products. This could be due to the generally poor reactivity of the chlorides [43,44]. Modifications in base, reaction time, solvent, or reaction temperature could not afford tetra-arylated tetracene. Remarkably, the Pd_2_(dba)_3_/XPhos catalyst system resulted in a drastic improvement in the yield of **3** to 72%. Here we anticipate that trialkylphosphines could also improve the yield instead of Xphos, although it was not chosen due to additional costs and its pyrophoric nature. After several screenings of solvent mixtures such as dioxane/water, dioxane/MeOH/water, THF/water, and MeOH/THF/water, etc., toluene/THF/water (3/1/1, *v*/*v*/*v*) was found to furnish the highest yield, with the least amount of unassignable impurities. In our finding, the most efficient protocol to synthesize tetra-arylated naphthalene or tetracene was the multiple Suzuki-Miyaura cross coupling performed on **1/6** in the presence of Pd_2_(dba)_3_/Xphos, Na_2_CO_3_, toluene/THF/water (3:1:1), and aryl boronic acids (Scheme 2). Furthermore, this method proved to be successful for the synthesis of **8** with a 71% yield, which was characterized with NMR, MS, UV-vis spectrophotometry, and SCXRD.

To note, the general method of rubrene synthesis is the treatment of 1,1,3-Triphenyl-2-propyn-1-ol with thionyl chloride [40]. Despite several instances in the literature supporting the utility of this method to synthesize symmetrically or asymmetrically substituted tetracenes, there is no report of its use for synthesizing tetrachalcogenophenyl-substituted tetracene. This could be associated with the unfavorable reactivity of the chalcogenophene rings with the radical that forms during this process. Contextually, the substrate scope in the Stille coupling method for tetra substitution on tetracene was limited to only incorporating chalcogenophenes [36]. The use of organoboronic compounds in Suzuki coupling could be environmentally more benign than the organostannyl derivatives used in Stille coupling, which is delivered by our method. The other methods for the synthesis of rubrenes involve the use of lithium or magnesium derivatives of aryl or alkynyl to react with the carbonyl centers of the quinoidal synthons of tetracene, followed by aromatization [45]. This organolithium/magnesium is known to react with several functional groups such as -CHO, -NH_2_, -NO_2,_ etc. (if already installed on other sites of the substrates) alongside the expected C=O groups, which limits the synthesis of such rubrenes carrying the aforementioned functional groups. Here we anticipate that the benefit of our protocol is the modification of the already aromatic tetrahaloacene core only at the final step, leaving scope for the synthesis of tetra-aryl-/heteroaryl-substituted tetracenes carrying even the lithium/magnesium sensitive functional groups for further extensions.

### 2.1. Morphology

**Figure 1:**
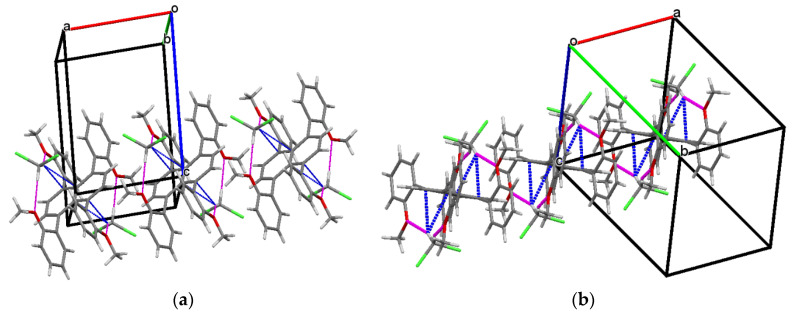
Packing arrangements of **3** along (**a**) c-axis and (**b**) a-axis. 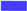 C-H···π interaction, 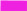 H-bonding interaction. The axes have been mentioned in the following manner. Red: a-axis, green: b-axis, blue: c-axis.

Single crystals of **3** were grown by a slow diffusion of methanol into its dichloromethane solution. The lattice was found to acquire a triclinic (P−1) space group, and the unit cell accommodated one molecule of **3** alongside two molecules of dichloromethane (Table S10). Some Peri-substituted naphthalenes, such as perchlorinated naphthalenes [46] or adamantyl-substituted equatorenes [47], enforce the twisting of the naphthalene core by more than 25° in the solid state. However, **3** carrying four *o*-anisyl groups showed planarity of the naphthalene backbone despite steric crowding from the four *o*-anisyl rings. In the single crystals diffracted at 123 K, the anisyl groups were found to be flanked at an angle of 170° from the plane of the naphthalene (∠C6-C2-C3 = 170°). The dihedral angle between the two peripheral *o*-anisyl rings (∠C13-C5-C2-C6 = −14.3°) on the same side of the acene core was −14.3°. Moreover, the presence of different hetero atoms (O, Cl) in the asymmetric unit opened pathways for intermolecular H-bonding between **3** and dichloromethane molecules (O1···H = 2.602 Å, O2···H = 2.412 Å) and several C-H···π interactions, (Figure 1) which served as additional packing forces. One typical example here is the interaction between H12a, C15, and C16 and the centroid of the rind (X) [48]. The distance between H12a and X is 2.66 Å. ω was found to be 69.69° between the planes ▱(C15-C16-H12A) - ▱(C15-C16-X) = 69.69 and θ was found to be constructed to be 9.47° (∠H12a-C12-C15 = 9.47) (Figure S9). So, the non-planar orientations of the o-anisyl substitutions around the naphthalene core and several other packing forces rendered **3** the planarity of the naphthalene core.

**Figure 2:**
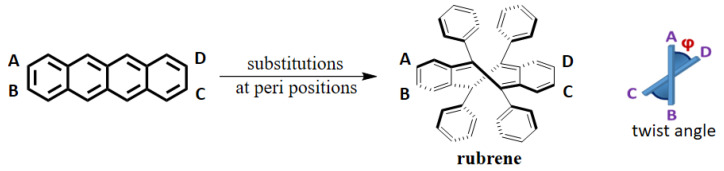
Schematic representation of introduction of twist to acene backbone and twist angle (*ϕ*). A,B,C and D are the C-atoms, through which the longitudinal twist angle has been calculated.

Among many discerning factors, morphology in the solid state has been a very important one because it modulates the charge transport in organic materials. The planar π-conjugated core of acenes that enterprises π-π stacking and rapid hopping of charge is still considered to be a suitable design principle for furnishing new organic electronic materials [49]. Rubrene has served as a benchmark material in organic electronics for its elegant photophysical and optoelectronic properties and exceptionally high hole mobility [50]. Although gas phase optimized (DFT/CAM-B3LYP/SDD), rubrene has a twisted tetracene backbone with a twist angle (*ϕ*) of 33° [49]. However, the SCXRD analysis confirms the planarity of the tetracene core in the solid state, thus facilitating π-π stacking in addition to the herringbone packing motif via C-H···π interaction in rubrene [51]. The planarity of the tetracene core in rubrene is believed to be adjusted by the non-planarity of the phenyl substituents around it, which forms a dihedral angle of 25° with the tetracene backbone. The DFT calculations performed by Beran et al. revealed that the twisting energy is only 8–10 kJ mol^−1^ (Figure 2) [52]. However, it could demand 40 kcal mol^−1^ to afford *ϕ* of 80° [53]. Notably, upon hydrostatic compression of rubrene at higher than 6.0 GPa, it was reported to undergo a structural change from a planar acene core to a doubly twisted acene core in compensation for 70 kJ/mol stabilization energy [54]. However, a small variation in substituting peripheral phenyl rings could result in the twisting of the acene core [9]. Nevertheless, no example was found with a twisted tetracene core in rubrene, even though many of its entries are in CCDC, which means predicting a twisted acene backbone in a solid state by looking at the molecular structure is not a straightforward problem.

**Figure 3:**
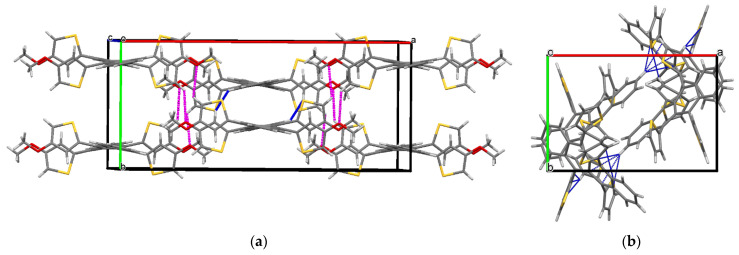
Packing arrangement along c-axis of (**a**) compound **8** and (**b**) 5,6,11,12-tetrathienylrubrene [30]. 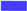 C-H···π interaction, 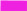 H-bonding interaction. The axes have been mentioned in the following manner. Red: a-axis, green: b-axis, blue: c-axis.

The single crystals of **8**, grown by diffusion using chloroform and methanol, appeared as pink plates. The SCXRD experiment operated at 123.5 K showed that **8** crystallized in a Pbcn space group (Table S10). The asymmetric unit consisted of one molecule of **8** with a helically twisted tetracene core in *M*-fashion providing a *ϕ* of −24.4°. The unit cell was found to accommodate four molecules via several non-covalent interactions. In the lattice, conglomerates of equal numbers of *P* and *M*-helices of **8** were found. One of the conglomerates had a slipped stack pattern with the terminal benzene rings placed in a cofacial orientation with ~3.5 Å (C2-C5 = 3.565 Å and C4-C7 = 3.508 Å) between them. This distance is shorter than the van der Waals distance of two C-atoms, which indicates strong π-π stacking in **8**. These closely packed conglomerates were found to be extended along the c-axis (Figure 3a). The driving force for this packing motif could be ascribed to the van der Waals interaction between the β hydrogen atom of the thiophenes and the H-atoms of the terminal benzene ring (H3···H16 = 2.39 Å). The incorporation of methoxy groups provides several pathways for packing. *First*, the intermolecular H-bonding between the O-atom and the α hydrogen atom (H13···O1 = 2.678 Å, H18···O2 = 2.707 Å) of the thiophenes forced face-to-face packing of *M*-**8** along the b-axis in the form of the conglomerates with 7.09 Å between them. *Second*, the C-H···π interaction between the H-atom of the –OMe groups and the π-electrons of the electron-rich thiophene rings (C11···H19B = 2.87 Å) extends the lattice in the form of agglomerates. Notably, the previously reported twisted 5,6,11,12-tetrathienylrubrene lacked any π-stacking, detrimental to its performance in OTFT [36]. So, the insertion of methoxy groups into the thiophenes resulted in a significant alteration in the morphology of 5,6,11,12-tetrathienylrubrene. This could garner a significant improvement in device performance for **8** when applied in relevant areas of organic electronics.

Generally, twisting an acene backbone sequentially induces significant optical activity, which could have applications as solid-state CPL emitters [55] and spin filters [26]. However, the twistacenes are not known to show π-π stacking in the solid state due to steric crowding of the acene cores, which could limit them in such device applications. Remarkably, **8** exhibited a twistacene core with π-π stacking. In line with this, Mamada et al. showed that any chalcogenophenyl substitutions at 5,6,11, or 12 positions of tetracene result in a twisted geometry of the acenes in solid state. Thus, in experiments that focus on solid-state optical activity, core-twisted acenes such as **8** could be more beneficial than planar compounds such as rubrene. However, chiral resolution of **8** could be unsuccessful, as the *M*- and *P*-helical **8** could easily overcome the barrier of racemization since there is no tether or any other driving force to restrict the conformational flipping [10,16]. However, the solid-state structure of **8** promises that a judicial design to modify this molecule into a helically locked acene could further pave the way for accessing its stable enantiomers in the solid state.

### 2.2. Experimental and Computational Photophysical Properties

**Figure 4:**
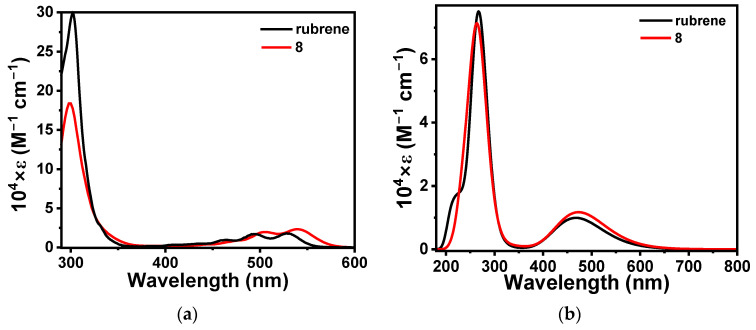
(**a**) Experimental UV-vis spectra of rubrene and **8** in chloroform and (**b**) simulated (DFT/CAM-B3LYP/SDD) UV-vis spectra at gas phase.

The UV-vis absorption spectra of **8** were recorded in chloroform solution and compared with newly synthesized rubrene under identical experimental conditions (Figure 4a). The spectral features of rubrene exhibited identical features, as reported earlier [49]. Compound **8** displayed two major bands inherent to the tetracenes. The vibronically allowed low energy band in the 450–550 nm region with a maximum at 540 nm could be ascribed to the ^1^L_a_ transition [56]. The HOMO-LUMO gap from the solution state spectra could be calculated as 2.23 and 2.16 eV for rubrene and **8**, respectively. So, the four 3-methoxythienyl substituents in **8** instead of phenyls alleviated the HOMO-LUMO gap by only 0.07 eV. The ^1^L_a_ band consisted of four clear vibronic shoulders at 446, 474, 500, and 540 nm. Remarkably, the vibronic shoulders were bathochromically shifted from the corresponding peaks of rubrene by 12 nm each. In addition, the smallest energy vibronic transition at 540 nm in the ^1^L_a_ band for **8** was found to have 1.2 times higher intensity than the nearest peak at 506 nm. In rubrene, these corresponding low-energy vibronic transitions were found to show similar intensity. The combination of these facts hints that the replacement of the four phenyl rings in rubrene with four 3-methoxythienyl rings in **8** does not alter the inherent vibronic pattern of the S_1_ state of tetracene but only alters the oscillator strength (*f*) for the lowest energy vibronic transitions. On the other hand, the higher energy band at 299 nm could be attributed to the typical ^1^B_b_ transition for the acenes [57]. The experimentally observed spectral pattern and the simulated (TDDFT/CAM-B3LYP/SDD) UV-vis spectra of rubrene and **8** complemented each other (Figure 4b).

### 2.3. Atomistic Tuning of Chiroptical Activity: Computational Analysis

**Figure 5:**
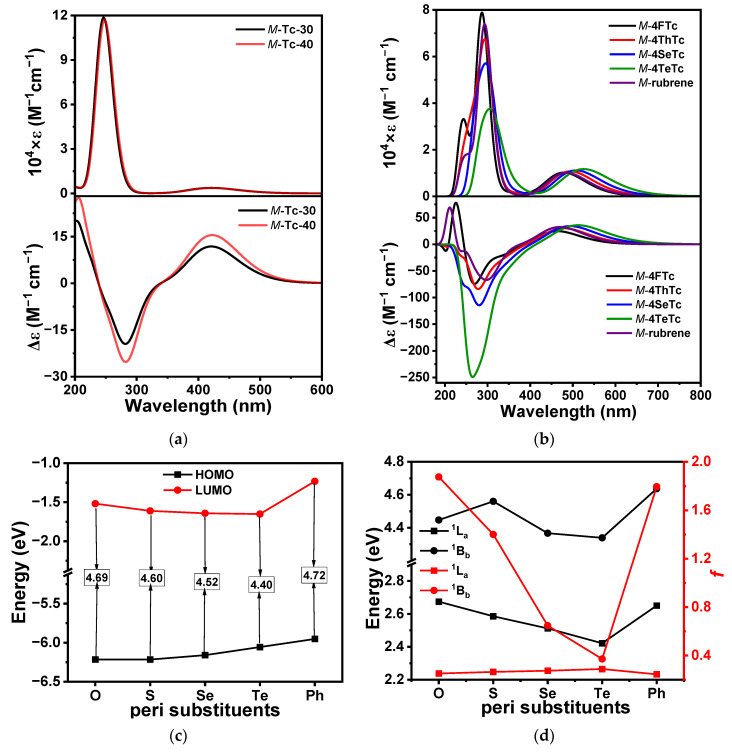
UV-vis (**upper panel**) and ECD spectra (**lower panel**) of optimized (**a**) twisted parent tetracenes, (**b**) tetraaryl tetracenes. (**c**) HLGs of the tetraaryl tetracenes. (**d**) Transition energies (black traces) and oscillator strength (*f*) (red traces) for the ^1^L_a_ and ^1^B_b_ transitions in tetraaryl-tetracenes.

The simulation supported the experimentally observed spectral features in the UV-vis spectrophotometry of the tetracenes. (Figure 5a) The computationally obtained absorption spectra of the tetracenes consisted of two major observable transitions, which are (a) the low-energy ^1^L_a_ (or *p* transition) corresponding to the HOMO-LUMO transition and (b) the ^1^B_b_ band (β transition) (Tables S3 and S4) [16]. This establishes the reliability of this calculation method for the series of molecules, where their chiroptical activities could be analyzed in a systematic manner. The synthesized twistacenes could not be isolated into their enantiomers due to the small barrier for conformational flipping. However, the computational analysis allows us to understand their chiroptical properties via an atomistic approach. For the convenience of systematic study, only the pure chalcogenophenyl-substituted tetracenes (*M*-**4FTc**, *M*-**4ThTc**, *M*-**4SeTc** and *M*-**4TeTc**) have been computed alongside *M*-rubrene and parent acenes (Table 1). However, the geometry for synthesized molecule **8** was optimized at the same level of theory (Table 1). Parent acenes were studied within the relevant limit of *ϕ* (between 30 and 40°) observed in the optimized heteroaryl-substituted tetracenes (Table S5). The role of the heteroatom in optical activity was not studied before this report. So, we have limited our study to the chalcogenophene substitutions around the acenes based on our successful synthesis of compound **8**. We found that atomistic tuning in the heteroaryl groups results in a bathochromic shift of the absorption spectra (Figure 5b). The energy required for the ^1^L_a_ transition was systematically decreased from 2.67 eV for *M*-**4FTc** to 2.42 eV for *M*-**4TeTc** and found a hyperchromic shift due to a systematic increase in *f* (Figure 5c,d). In contrast, the ^1^B_b_ transition found a red shift of 0.11 eV between the two. The molar extinction coefficient (*ε*) for the ^1^B_b_ transition was found to decrease rapidly from *M*-**4FTc** to *M*-**4TeTc**, while that for the ^1^L_a_ band was elevated slowly. The reason for the hypochromic shift of the ^1^B_b_ transition could be ascribed to the drastic decrease in *f* from *M*-**4F-Tc** to *M*-**4TeTc** (Figure 5d).

The chiroptical properties of the compounds at the gas phase geometry could also be assessed from the Cotton effects observed in the electronic circular dichroism (ECD) spectra. In the ECD spectra, the ^1^L_a_ and ^1^B_b_ transitions were found to show opposite signal polarity, similar to twisted parent tetracenes [16]. Generally, the ^1^L_b_ transition in tetracenes that appears in the region of ~350 nm is overshadowed by the much stronger ^1^B_b_ and ^1^L_a_ transitions, which is also evident in Figure 5b. Our computational calculation revealed that the Cotton effect could be atomistically tuned through the systematic alteration of the heteroatoms in chalcogenophenyl rings. At the same level of calculations, rubrene was found to have the weakest Cotton effect among all twistacenes in the shorter wavelength region, whereas that for the longer wavelength region was higher than *M*-**4FTc** and *M*-**4ThTc** but lower than *M*-**4SeTc** and *M*-**4TeTc**. Atomistic tuning of the heteroaryls down the chalcogen group resulted in the systematic increase in the *ϕ* of the acene core, which is also known to increase chiroptical activity independently [16]. However, this change in *ϕ* was only 8° from *M*-**4FTc** to *M*-**4TeTc**. Minor changes in *ϕ* are known to offer negligible alteration in the electronic and chiroptical properties of parent acenes [16]. On top of that, *M*-**4TeTc** exhibited a 2.3-fold increase in Cotton effect compared to its parent twistacene analog, *M*-**Tc-40**. This established that the significant increase in chiroptical activity exhibited by the present series of molecules is due to the alteration in chalcogens.

**Figure 6:**
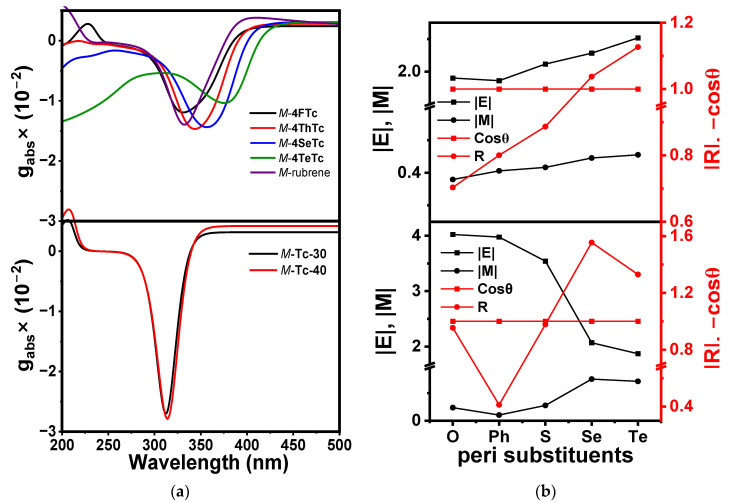
(**a**) *g*_abs_ spectra of heteroaryl-substituted tetracenes (**upper panel**) and twisted parent acenes (**lower panel**) and (**b**) transition dipole moment (TDM), rotatory strength (R) and cosθ of ^1^L_a_ (**upper panel**) and ^1^B_b_ (**upper panel**) and transitions.

To further understand these trends in the Cotton effect and *g*_abs_ (Δ*ε*/*ε*) (Figure 6a), transition dipole moment (TDM) analyses for the brightest transitions in the ECD spectra were performed (Figure 6b, Tables S2 and S7). The ECD of a given transition is proportional to its rotatory strength (R) = |E||M| cosθ, where |E| and |M| are the electric and magnetic transition dipole moments, respectively, and θ is the angle between them. |E| for the ^1^L_a_ transition provided very similar values for rubrene and *M*-**4FTc**, whereas |M| was significantly higher for *M*-**4FTc**. Both the |E| and |M| increased with the heavier heteroatoms in the chalcogenophenes. Like the parent acenes, the TDMs for the twistacenes were found to align in an antiparallel fashion, maximizing the value of the cosθ. These resulted in a systematic increase in the R of the ^1^L_a_ transition. On the other hand, the interplay of |E| and |M| was antagonistically systematic for the ^1^B_b_ transitions. We have considered the brightest states contributing to the UV-vis maxima in this wavelength region. |E| showed a 2-fold decrease from *M*-**4FTc** to *M*-**4TeTc**, while |M| increased by three times, and the cosθ remained at its maximum value. This resulted in a significant increase in R (Figure 6b). Although the increase in the Cotton effect of all the transitions was evident in the ECD spectra, remarkably, the factor was larger than 3.4 times for the ^1^B_b_ transition in *M*-**4TeTc** compared to *M*-**4FTc** or *M*-rubrene. Notably, the highest *g*_abs_ for the twistacenes originated at similar spectral positions of the ^1^L_b_ transition as the helically locked twisted anthracenes [16]. The *g*_abs_ increased systematically from *M*-**4FTc** to *M*-**4SeTc**, while it showed the lowest value for *M*-**4TeTc** (Figure 6a). However, the lack of a bright state in this spectral region and several states of weak oscillator strengths in the ~350 nm range restrict further unambiguous discussion on it.

## 3. Materials and Methods

Commercially available reagents and chemicals were used without further purification unless otherwise stated. Phenylboronic acid (**7**) was purchased from Sigma-Aldrich. Column chromatography was performed using silica gel (100–200 mesh). The ^1^H and ^13^C NMR spectra were recorded on Bruker AVIII 400 MHz and 500 MHz spectrometers using tetramethylsilane (TMS) as an external standard added to chloroform-d. Chemical shifts are expressed in *δ* (ppm) units. UV-vis absorption spectra were recorded using an Agilent Cary-5000 spectrophotometer. The spectra were measured using a quartz cuvette (1 cm) at 25 °C. High-resolution mass spectra were measured on an HR Q-TOF LCMS and the Waters Micromass GCT Premier Mass Spectrometer using ESI in a positive mode. The crystal structure was solved using Olex2 [57], with the SHELXT [58] structure solution program using intrinsic phasing, and refined with the SHELXL [59] refinement package using least squares minimization. The photophysical properties of twisted parent tetracenes and substituted tetracenes were investigated computationally by performing geometry optimizations and TD-DFT calculations using the CAM-B3LYP/SDD level of theory.

## 4. Conclusions

In summary, we have reported an efficient synthetic protocol for installing four peri-substitutions on naphthalene and tetracene in a single step by Pd(0)-catalysed multiple C-C cross-coupling reactions with high yields. The solid-state structure of 8 exhibited core twisted acenes of opposite helicities, which formed two types of conglomerate pairs, unlike planar rubrene. In one of those, the twistacenes are packed in a slipped stack motif, with the closest interplanar distance being shorter than the van der Waals radii. The –OMe groups in 8 were found to anchor some important packing motifs in a solid state, such as π-π stacking, C-H···π interaction, and H-bonding. The experimental and computational UV-vis spectra of 8 and newly synthesized rubrene complemented each other. The computational calculation revealed that the lowering of the HOMO-LUMO gap and systematic increase in the chiroptical activity could be achieved via atomistically tuning the chalcogenophene substitution around the main acene core. The differential peri-substitution could cause a minor change in *ϕ* of the acenes, but that adds a trivial contribution towards the manifold increase in optical activity, which arises mostly due to the variation in heteroaryls. ECD spectra showed that the Cotton effect of the chalcogenophenyl-substituted tetracenes in the shorter wavelength range was higher than that of the parent acenes and rubrene. For the HOMO-LUMO transition, the Cotton effect for rubrene is higher than *M*-**4FTc** and *M*-**4ThTc** and lower than *M*-**4SeTc** and *M*-**4TeTc**. So, this current report on computational studies on the chiroptical activity of 5,6,11,12-chalcogenophenyl-substituted twisted tetracenes and a new efficient synthetic protocol to access such molecules could be beneficial for designing chiral organic electronic materials, where helical solid-state morphologies are the prerequisites.

## Figures and Tables

**Table 1:** Optimized structures of the twistacenes at DFT/CAM-B3LYP/SDD level of theory.

	Front View	View through the Axis of Helicity	Side View	*ϕ* (°) ^a^	*ϕ* (°) ^b^
*M*-rubrene	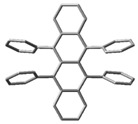	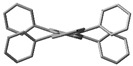	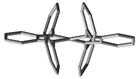	−33	0
*M*-**4FTc**	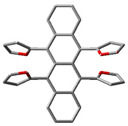	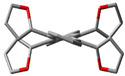	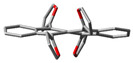	−33	27.8
*M*-**4ThTc**	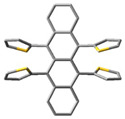	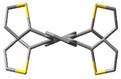	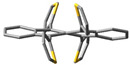	−37	29.1
*M*-**8**	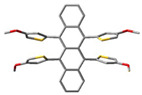	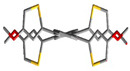	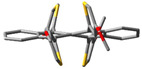	−33	− 24.4
*M*-**4SeTc**	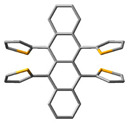	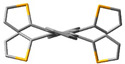	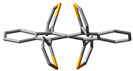	−39	-
*M*-**4TeTc**	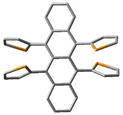	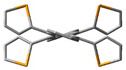	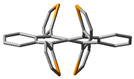	−41	-

^a^ Twist angle calculated from gas phase optimization. ^b^ Twist angle observed from crystal structure.

## Data Availability

Not applicable.

## Supplementary Materials

The following supporting information can be downloaded at: https://www.mdpi.com/article/10.3390/molecules28135074/s1. The detailed synthetic procedure, characterizations, computational methodologies and crystallographic information tables and files could be found here.

## Sample Availability

Not available.
