# Supplementary material for: Effect of Chalcogenophenes on Chiroptical Activity of Twisted Tetracenes: Computational Analysis, Synthesis and Crystal Structure Thereof
Source: Molecules. 2023 Jun 28;28(13):5074. doi: 10.3390/molecules28135074 (PMC10343419; doi:10.3390/molecules28135074)
Supplement: Supplementary file 1 [file molecules-28-05074-s001.zip › molecules-2456837-supplementary.pdf]

Supporting Information

Effect of chalcogenophenes on chiroptical activity of twisted tetracenes: Computational analysis, synthesis and crystal structure thereof

Gayathri J. K.,[1] Benny Bogoslavsky,[2] Sashi Debnath,[3] Anjan Bedi*[1]

[1] Department of Chemistry, SRM Institute of Science and Technology, Chennai, Kattankulathur, India, 603203

[2] Institute of Chemistry, The Hebrew University of Jerusalem, Edmond J. Safra Campus, Jerusalem, Israel, 91904

[3] Department of Radiology, University of Texas Southwestern Medical Center, Dallas, TX 75390, USA

* Correspondence: anjanb@srmist.edu.in

Contents

S1 General 2
S2 Synthesis 2
S3 Characterization 5
S4 Computations 10
S5 Analysis of C–H···π interaction 16
S6 Optimized coordinates of the tetraaryl-substituted tetracenes (DFT-CAMB3LYP-SDD). 19
S7 Optimized coordinates of the twisted parent tetracenes (DFT-CAMB3LYP-SDD) 26
S8 Reference 28

S1 General

General synthetic routes for tetraaryl-substituted acenes

Method A. Tetrahalocenes, aryl boronic acids (5.0 eq.), $Pd(PPh_3)_4$ (0.05 eq.), and Na_2CO_3 (6 eq.) were added to an argon-purged mixture of 1,4-dioxane and water (4:1). The resulting reaction mixture was heated to 90°C for 30 h. After cooling to room temperature, an excess of water was added; the organic layer was separated, and the aqueous layer was extracted with chloroform. The organic and aqueous layers were washed, dried over $MgSO_4$, and evaporated. The resulting residue was purified by Silica gel chromatography using ethyl acetate/hexane (1:4) as the eluent to afford a series of tetraaryl-substituted acenes (22%).

Method B. Tetrahaloacenes, aryl boronic acids (5.0 eq), $Pd_2(dba)_3$ (0.05 eq), XPhos (0.2 eq), and Na_2CO_3 (5.0 eq.) were added to an argon-purged mixture of THF/toluene/water (3:2:1). The resulting reaction mixture was heated to 95°C for 13 h. After cooling to room temperature, an excess of water was added; the organic layer was separated, and the aqueous layer was extracted with chloroform. The organic and aqueous layers were washed, dried over $MgSO_4$, and evaporated. The resulting residue was purified by Silica gel chromatography using ethyl acetate/hexane (1:4) as the eluent to afford a series of tetraaryl-substituted acenes (70-72%).

S2 Synthesis

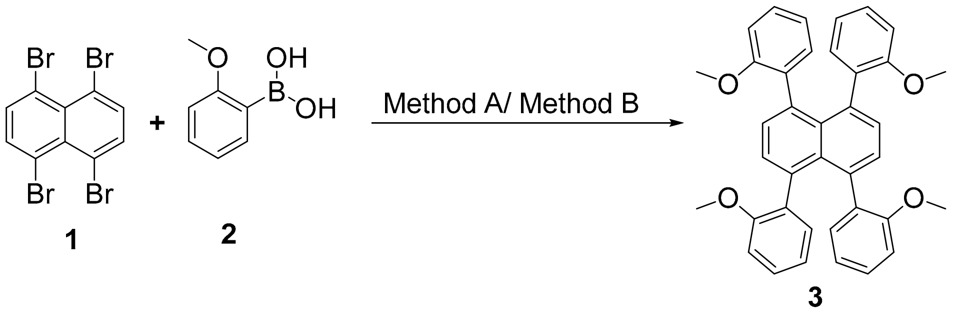

Scheme S1. Synthesis of tetraanisyl-substituted naphthalene. Conditions: method A- Pd $(PPh_3)_4$, Na_2CO_3, 1,4-dioxane: water (1:1), Ar, 90°C, 30 h, 22%. method B-$Pd_2(dba)_3$, XPhos, Na_2CO_3, toluene/THF/water (3:2:1), Ar, 95°C, 13 h, 72%.

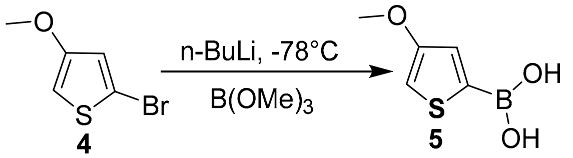

Scheme S2. Synthesis of **5**.

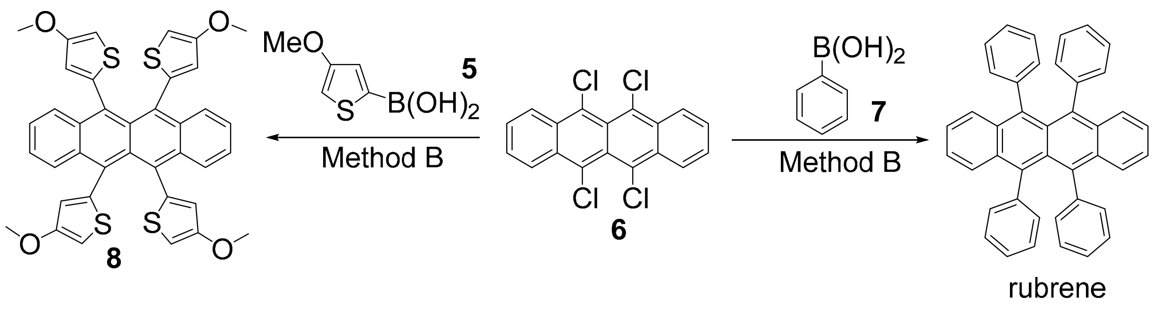

Scheme S3. Synthesis of tetraaryl-substituted tetracenes. Conditions: Method B. $Pd_2(dba)_3$, X-Phos, Na_2CO_3, toluene/THF/water (3:2:1), Ar, 95°C, 13 h, 70%.

S2.1 Synthesis of **3**.

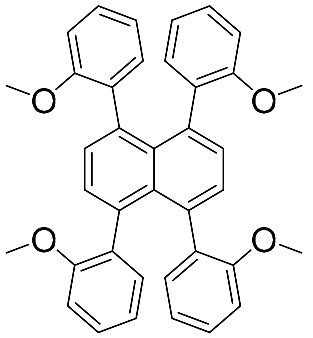

Method A. Compound **3** was obtained as a white solid (0.028 g, 22%) from **1** (0.100 g, 0.225 mmol), **2** (0.171 g, 1.125 mmol), Pd $(PPh_3)_4$ (0.013 g, 0.011 mmol), and Na_2CO_3 (0.143g, 1.350 mmol).

Method B. Compound **3** was obtained as a white solid (0.125 g, 72%) from **1** (0.140 g, 0.315 mmol), **2** (0.287 g, 1.890 mmol), $Pd_2(dba)_3$ (0.014 g, 0.016 mmol), XPhos (0.030 g, 0.063 mmol), and Na_2CO_3 (0.167 g, 1.575 mmol).

^{1}H NMR (400 MHz, $CDCl_3$) δ 7.20 (d, *J* = 2.0 Hz, 4H), 7.08 (dd, *J* = 7.4, 1.8 Hz, 4H), 6.93 – 6.88 (m, 4H), 6.73 – 6.66 (m, 4H), 6.29 (d, *J* = 8.2 Hz, 4H), 3.56 (d, *J* = 5.8 Hz, 12H).

HR-ESI-MS m/z (%): calcd. for $C_{38}H_{33}O_4$ ($[M+H]^+$), 553.2379; Found 553.2364.

S2.2 Synthesis of **5**.

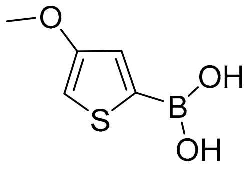

In an oven dried Schlenk tube, **4** (2.500 g, 12.949 mmol) was diluted in anhydrous THF. The flask was cooled at –78 °C under dry Ar, and *n*-BuLi (1.6 M, 7.9 mL, 12.642 mmol) was added. The resulting reaction mixture was stirred at –78 °C for 1 h, followed by the dropwise addition of $B(OMe)_3$ (1.342 g, 12.896 mmol), and then allowed to warm to room temperature after 0.5 h. The reaction mixture was treated with 2M HCl (15 mL, 30 mmol), extracted using ethyl acetate. The organic extract was dried and concentrated in rotavapor to result in a white solid (480 mg, 40%). Thus, obtained analytically, the pure crude solid was directly used for the next step without further purification.

^1H NMR (400 MHz, $CDCl_3$) δ 7.56 (d, J = 1.5 Hz, 2H), 6.75 (d, J = 1.5 Hz, 2H), 3.87 (s, $B(OH)_2$, 2H). HR-ESI-MS m/z (%): calcd. for $C_5H_8BNaO_3S$ ($[M+Na]^+$), 181.0107; Found 181.0141.

S2.3 Synthesis of **rubrene**.

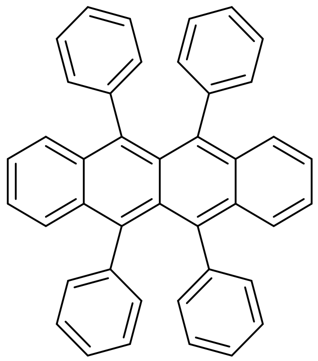

Method B. Rubrene was obtained as a red solid (0.102 g, 70%) from **6** (0.100 g, 0.273 mmol), **7** (0.166 g, 1.365 mmol), $Pd_2(dba)_3$ (0.012 g, 0.014 mmol), XPhos (0.026 g, 0.055 mmol), and Na_2CO_3 (0.145 g, 1.365 mmol).

^1H NMR (400 MHz, $CDCl_3$) δ (ppm) 7.36 (1H, m), 7.11 (2H, m), 7.05 (2H, m), 6.86 (2H, m). ^{13}C NMR (126 MHz, $CDCl_3$) δ (ppm) 140.21, 135.46, 130.57, 128.7, 127.56, 125.63, 124.17, 123.28, 75.43.

S2.4 Synthesis of **8**.

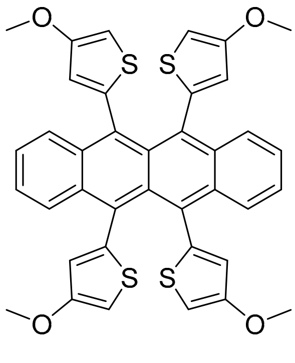

Method B. Compound **8** was obtained as a pink solid (0.288 g, 71%) from **6** (0.222 g, 0.606 mmol), **5** (0.479 g, 3.030 mmol), $Pd_2(dba)_3$ (0.028 g, 0.030 mmol), XPhos (0.058 g, 0.121 mmol), and Na_2CO_3 (0.321 g, 3.030 mmol).

^{1}H NMR (400 MHz, $CDCl_3$) δ (ppm) 7.78 (4H, m), 7.29 (4H, m), 3.88 (12H, s).
^{13}C NMR (126 MHz, $CDCl_3$) δ (ppm) 158.1, 140.7, 131.7, 131, 129.8, 129, 128.4, 126.3, 122, 77.0.
HR-ESI-MS m/z (%): calcd. for $C_{38}H_{29}O_4S_4$ ($[M+H]^+$), 677.0949; Found 677.0925.

S3 Characterization

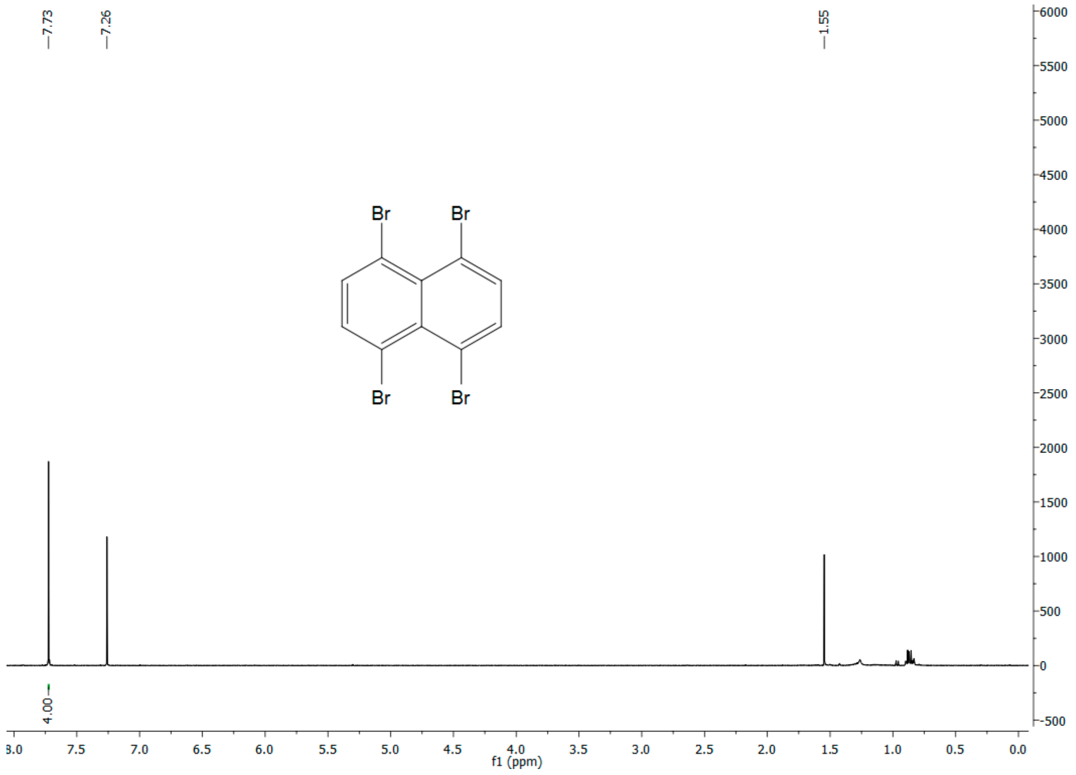

Figure S1. ^{1}H NMR of compound **1** at 298 K in $CDCl_3$.

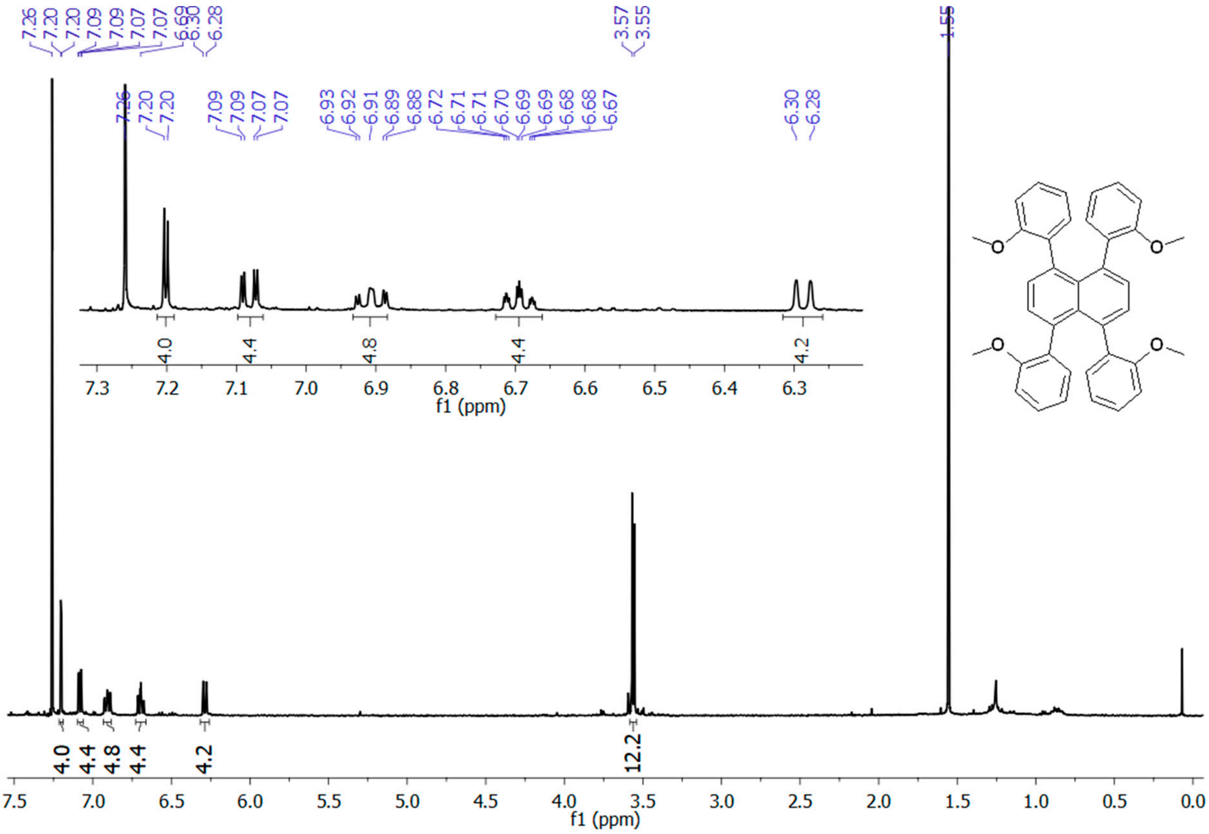

Figure S2.^{1}H NMR of compound **3** at 298 K in $CDCl_3$.

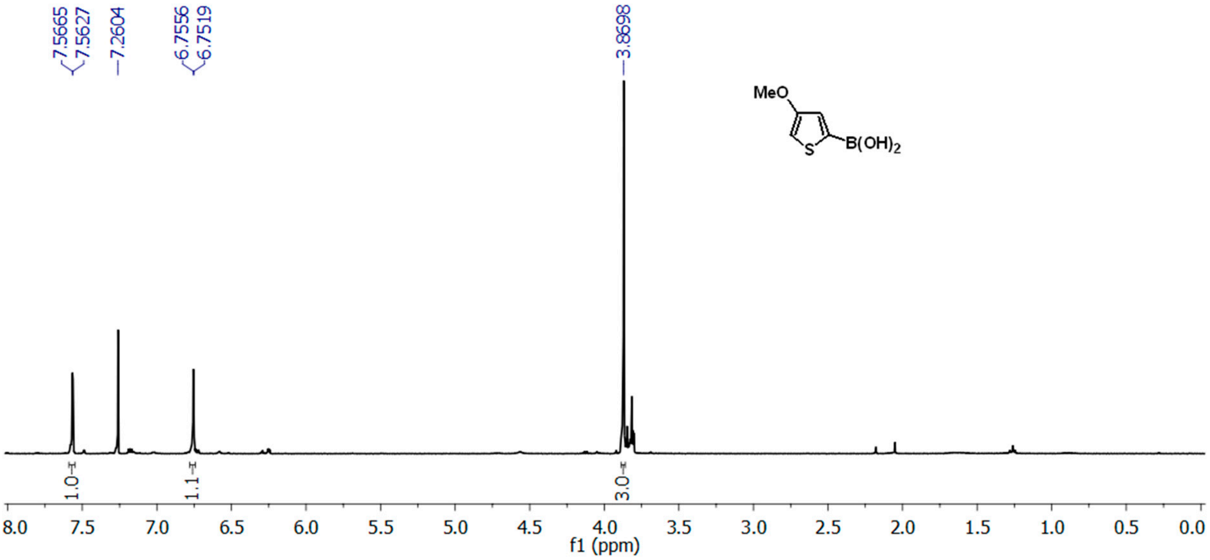

Figure S3. ^{1}H NMR of compound **5** at 298 K in $CDCl_3$.

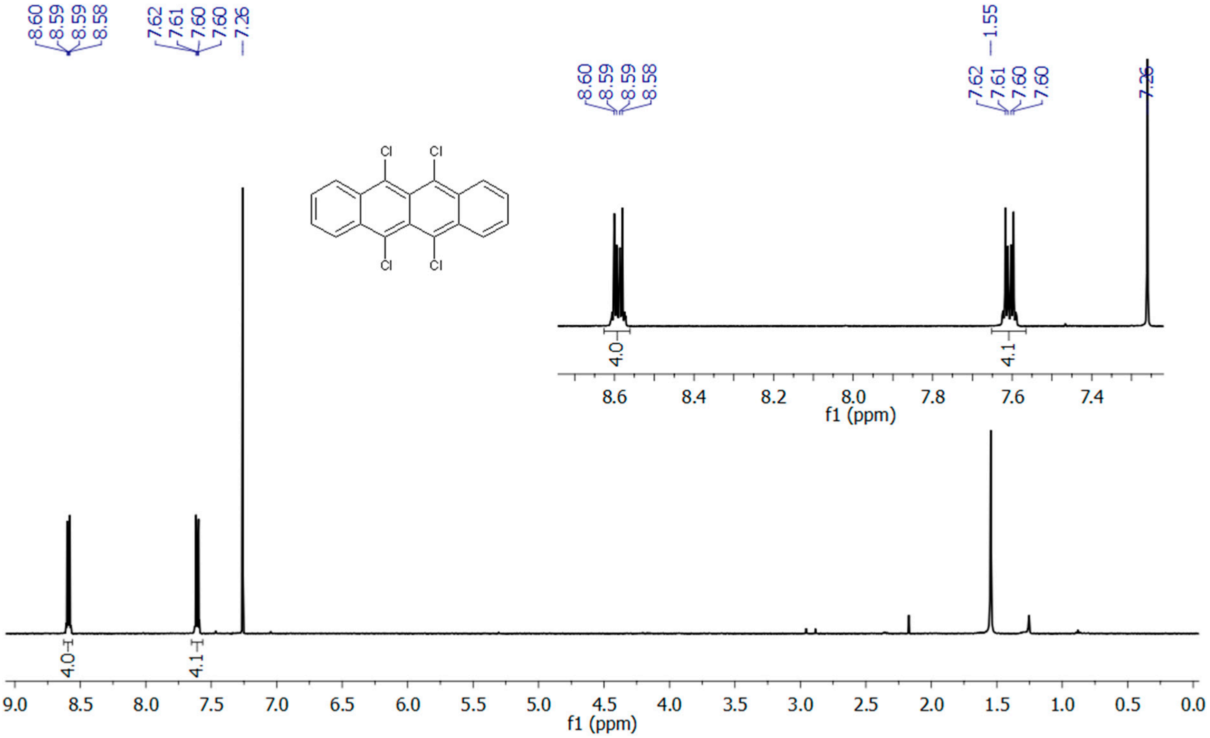

Figure S4. ^{1}H NMR of compound **6** at 298 K in $CDCl_3$.

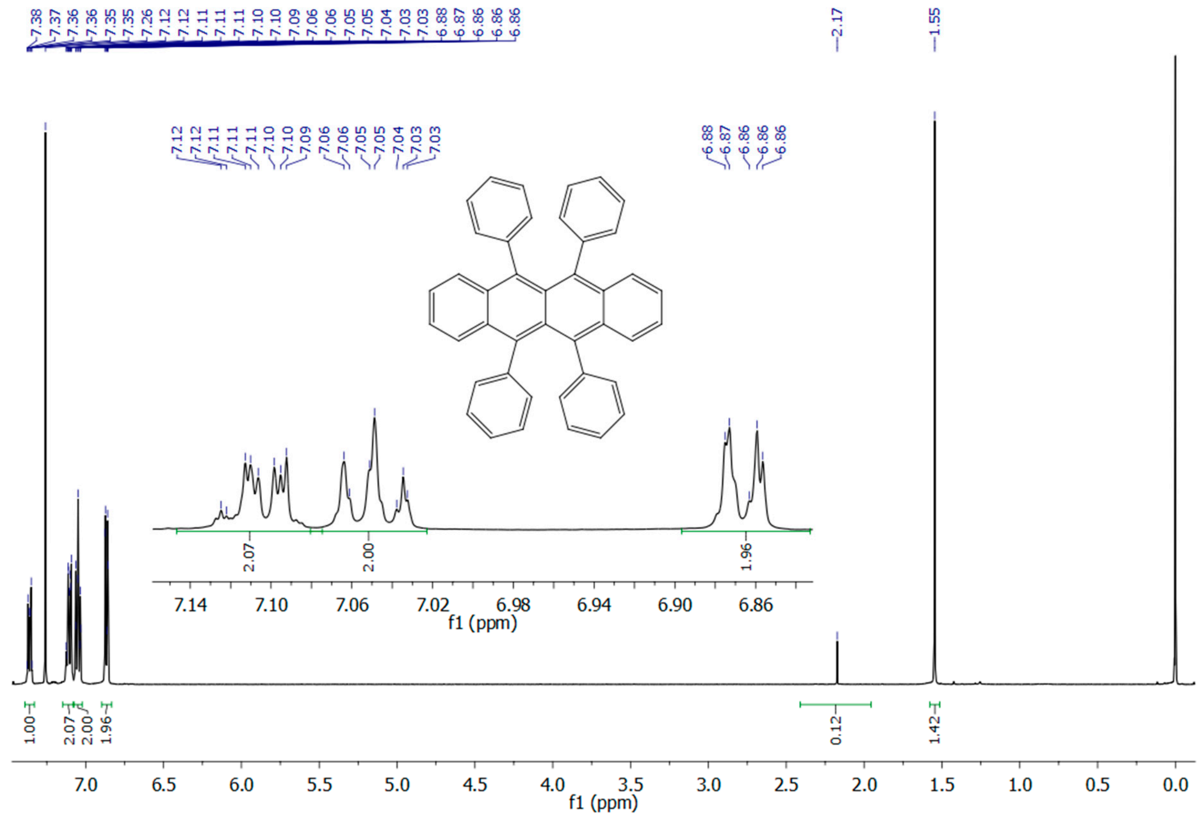

Figure S5. ^{1}H NMR of rubresne at 298 K in $CDCl_3$.

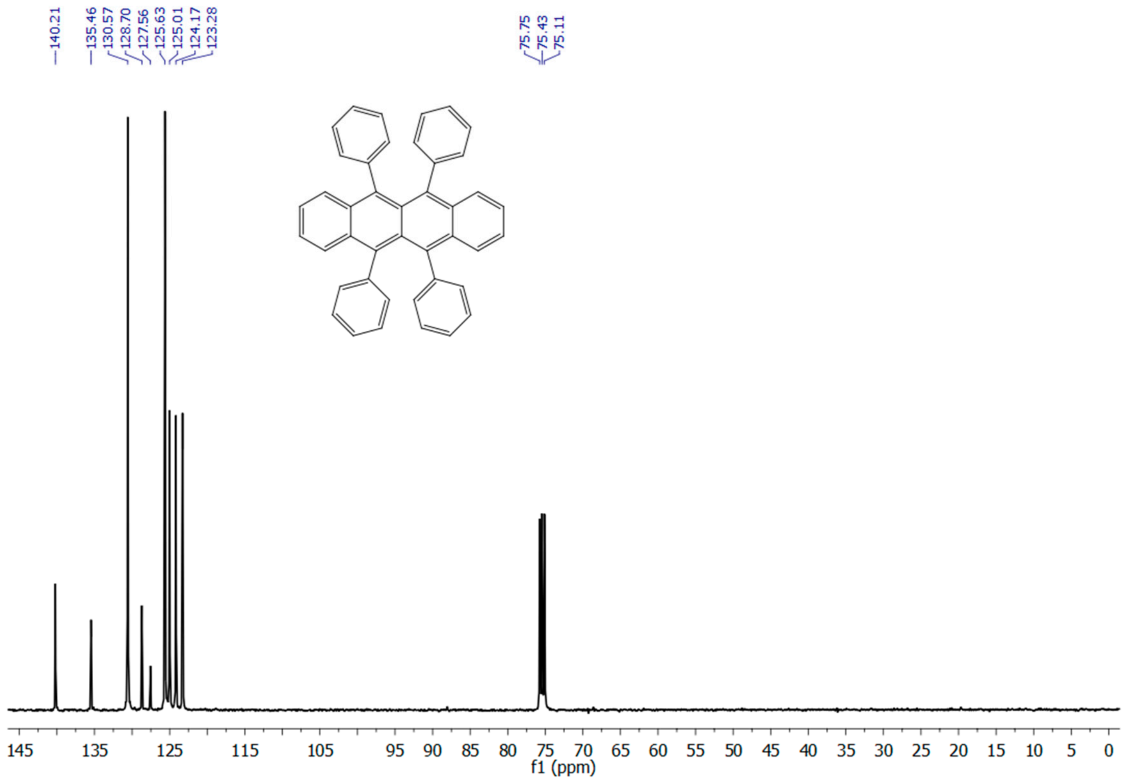

Figure S6. ^{13}C NMR of rubrene at 298 K in $CDCl_3$.

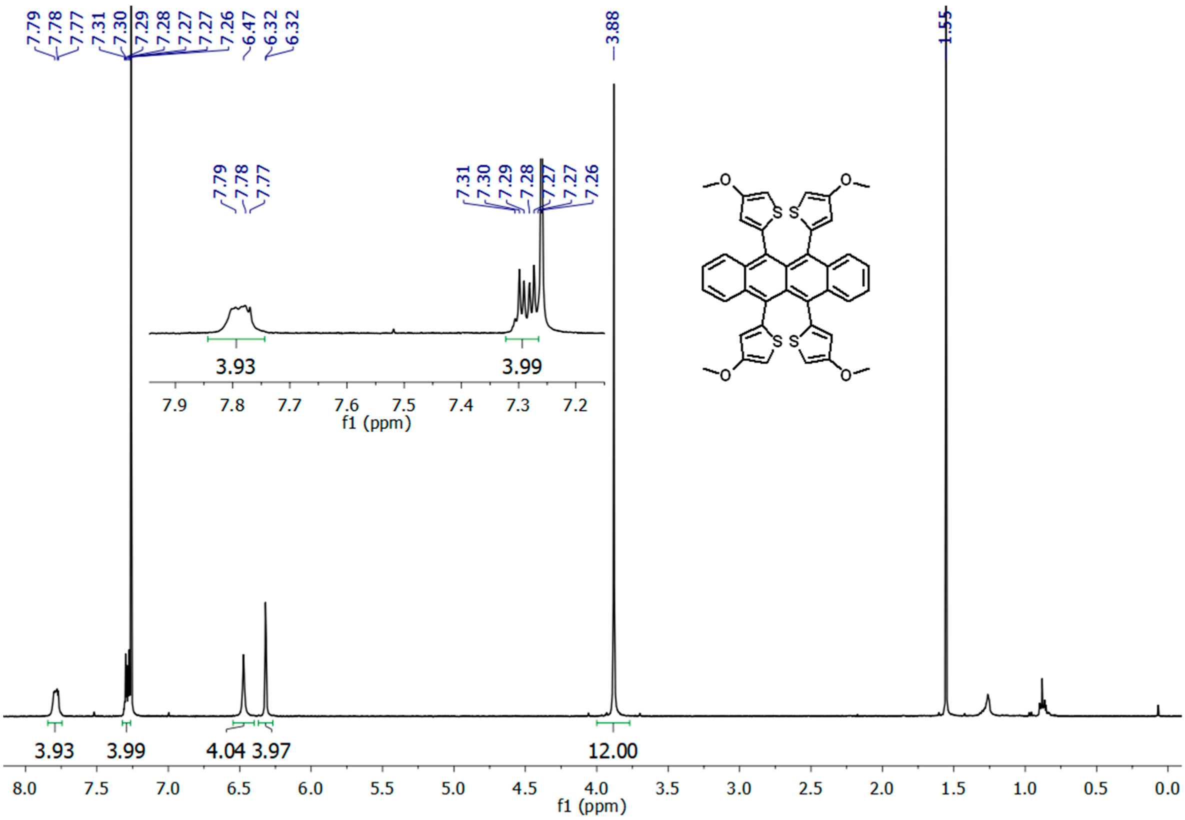

Figure S7. ^{1}H NMR of compound **8** at 298 K in $CDCl_3$.

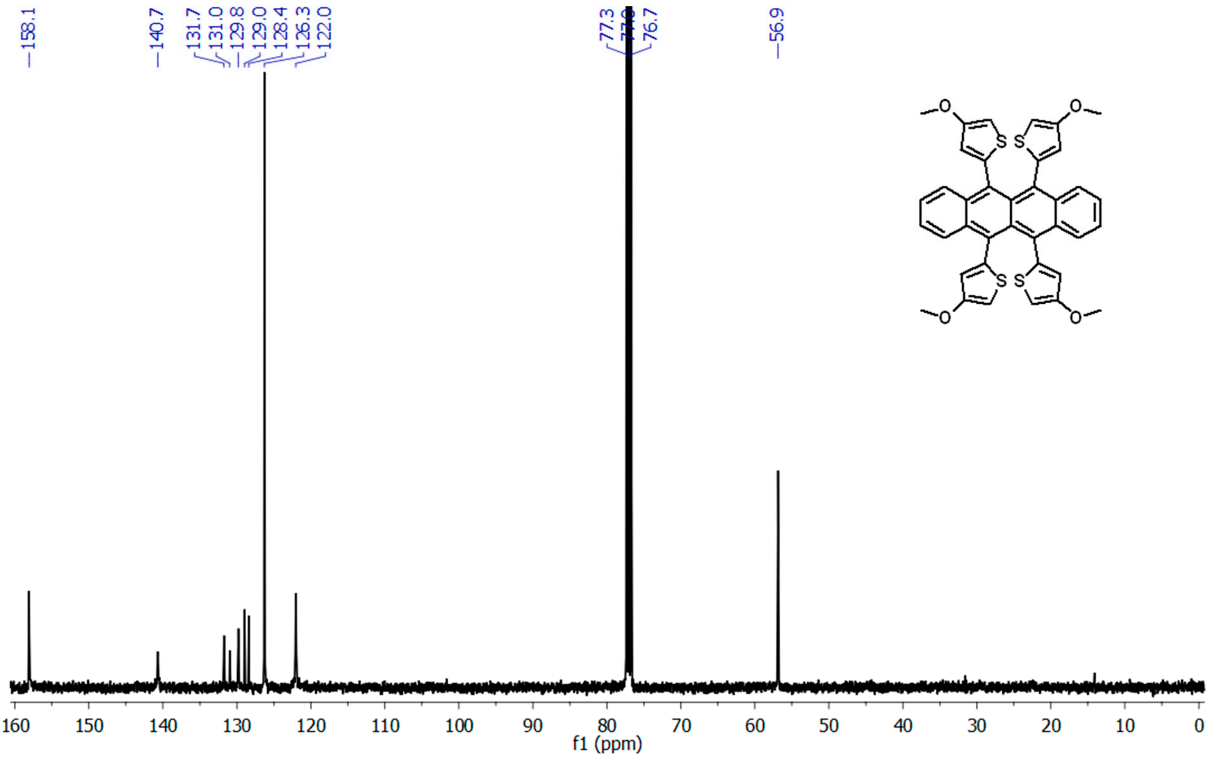

Figure S8. ^{13}C NMR of compound **8** at 298 K in $CDCl_3$.

S4 Computations

S.4.1. Details of computational calculations

All the computations were performed using density functional theory (DFT) and the Gaussian 16 program, Revision A. 03. In our earlier study, the use of the same functional on 6-31G(d) basis set efficiently complemented the experimentally observed Cotton effect in twistacenes, which proved to be superior to the M06-2X level of calculations as well. So, here we have used the identical functional in combination with SDD basis set to avoid any underestimation in the approximation arising from the presence of heavier atoms in current series of molecules.[1]

Table S1. Absolute energies of tetraaryl-substituted tetracenes optimized (DFT-CAMB3LYP/SDD) of tetraaryl-substituted tetracenes.

	Energy (Hartree)
M-**4FTc**	−3196.7211734
M-**4ThTc**	−3157.4012014
M-**4SeTc**	−3118.0869797
M-**4TeTc**	−3078.7721327
M-**rubrene**	−1616.1235772

S.4.2 Analysis of the optical transitions of tetraaryl-substituted tetracenes

Table S2. Electric (green) and magnetic (purple) transition dipole moments for the ^1L_a and ^1B_b transitions of tetra-aryl substituted tetracenes.

	^1L_a	^1B_b
***M*-4FTc**	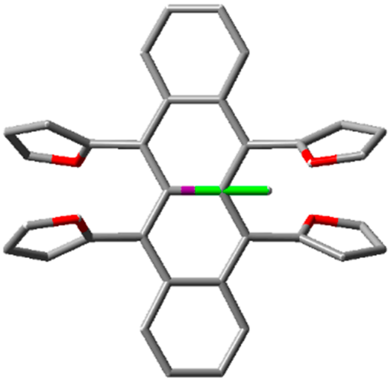	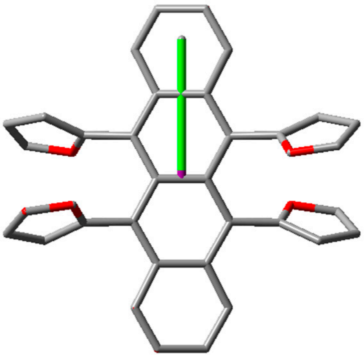
***M*-4ThTc**	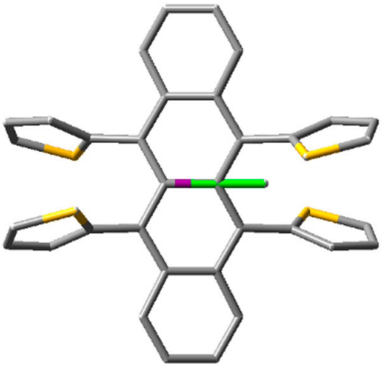	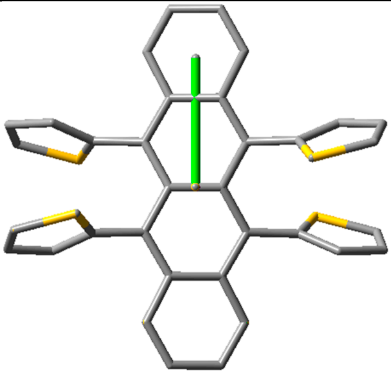
***M*-4SeTc**	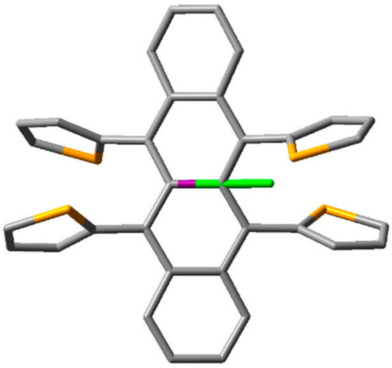	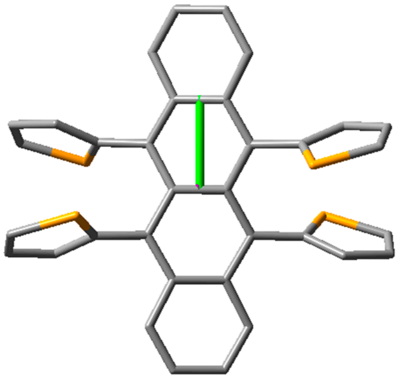
***M*-4TeTc**	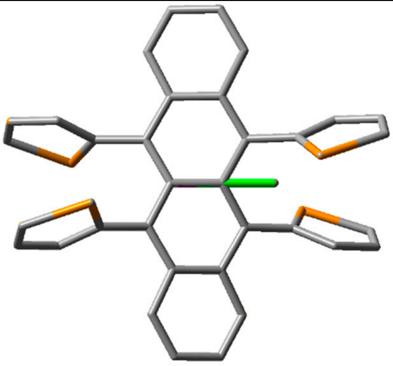	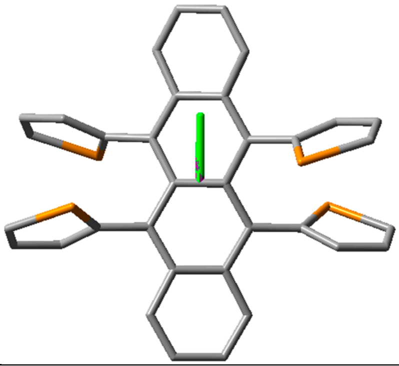

M-**rubrene**	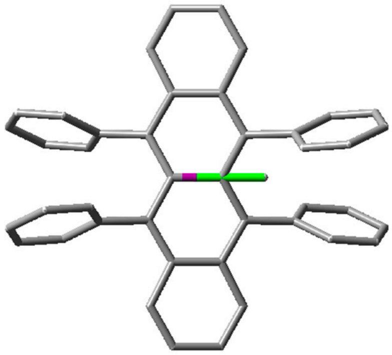	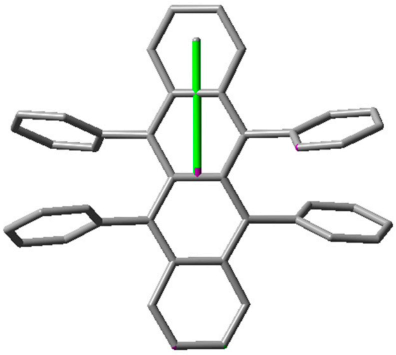

Table S3. Molecular orbitals (MOs) of the tetra-aryl substituted tetracenes involved in ^1L_a and ^1B_b electronic transitions.

	**LU+1**	**HOMO**	**LUMO**
**4FTc**	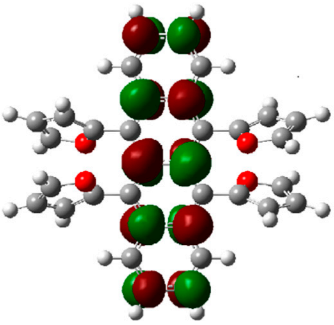	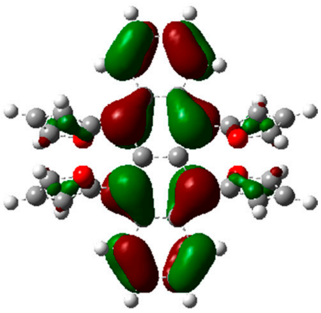	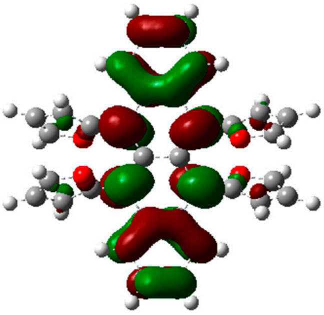
**4ThTc**	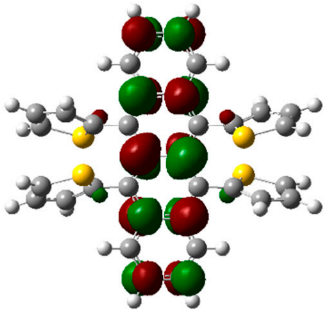	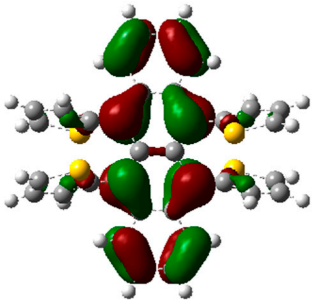	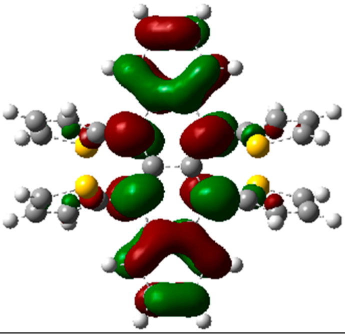
	**HO-6**	**HOMO**	**LUMO**
**4SeTc**	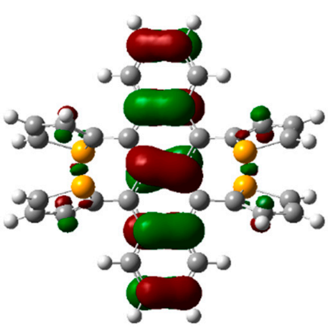	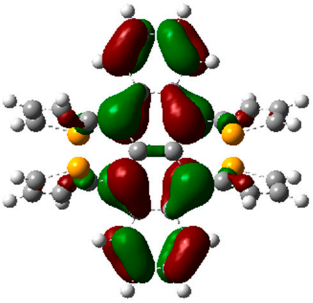	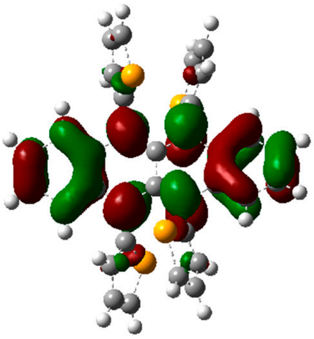
	**HO-8**	**HOMO**	**LUMO**

**4TeTc**	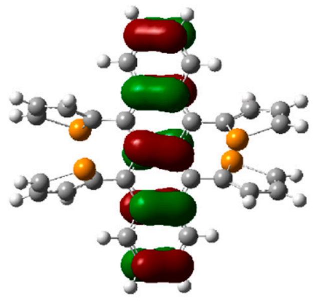	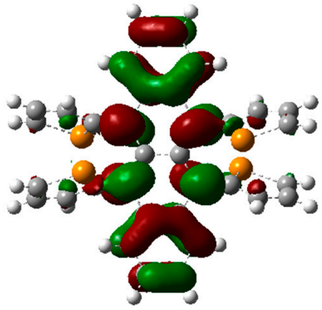	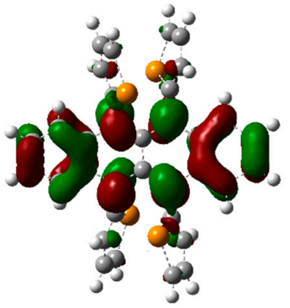
	**LU+1**	**HOMO**	**LUMO**
**rubrene**	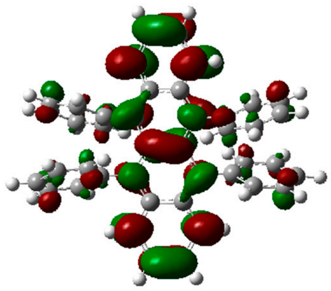	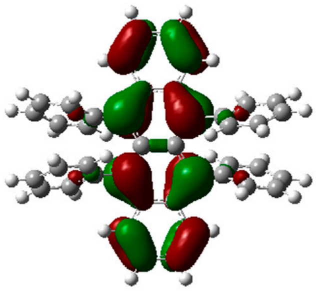	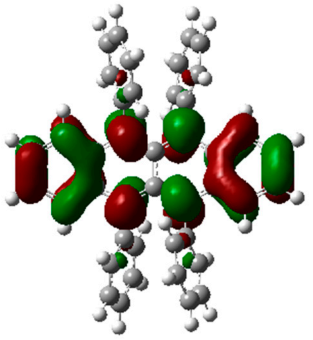

Table S4. Calculated lowest energy transitions for tetraaryl-substituted tetracenes and their orbital contribution. The brightest state has been considered for $^{1}B_{b}$ transitions.

	State number	Oscillator Strength (*f*)	Transition energy (eV)	Major transitions			
				Orbitals involved	Contri bution (%)	Orbitals involved	Contri bution (%)
***M*-4FTc**	**1**	**0.2518**	**2.6743**	**HO→LU**	**99.35**		
	**9**	**1.8752**	**4.4469**	**HO→LU+1**	**54.35**	HO-3→LU	39.37
***M*-4ThTc**	**1**	**0.2652**	**2.5858**	**HO→LU**	**99.42**		
	**10**	**1.4007**	**4.5602**	**HO-1→LU**	**48.33**		
***M*-4SeTc**	**1**	**0.2749**	**2.5121**	**HO→LU**	**99.41**		
	**10**	**0.6473**	**4.3669**	**HO-6→LU**	**41.93**	HO→LU+1	30.72
***M*-4TeTc**	**1**	**0.2891**	**2.4218**	**HO→LU**	**99.15**		
	**18**	**0.3718**	**4.3389**	**HO-8→LU**	34.71	HO→LU+6	16.81
***M*-rubrene**	**1**	**0.2454**	**2.6503**	**HO→LU**	**99.23**		
	**8**	**1.7951**	**4.6369**	**HO→LU+1**	**42.16**	**HO-4→LU**	**38.39**

Table S5. Optimized structures of twisted parent tetracene.

Twist angle (°)	Front view	Side view
30	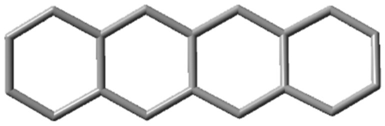	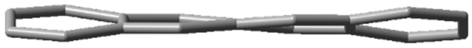
40	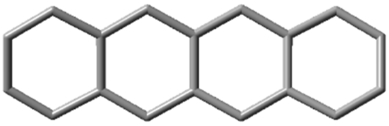	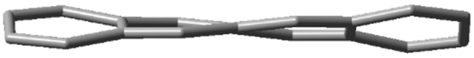

Table S6. Absolute energies of optimized (DFT-CAMB3LYP/SDD) twisted parent tetracene.

Twist angle (°)	Energy (Hartree)
30	-692.6359049
40	-692.6315853

S.4.3 Analysis of optical transitions of twisted parent tetracene

Table S7. Electric (green) and magnetic (purple) transition dipole moments for the ^1L_a and ^1B_b transitions of twisted tetracenes.

Twist angle (°)	^1L_a	^1B_b
30	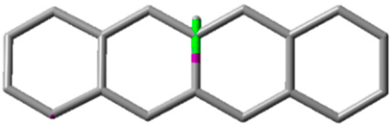	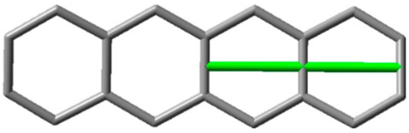
40	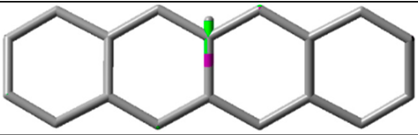	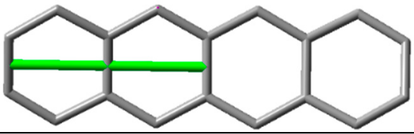

Table S8. Molecular orbitals (MOs) of the twisted parent tetracenes involved in ^1L_a and ^1B_b electronic transitions.

Twist angle (°)	LU+1	HOMO	LUMO
30	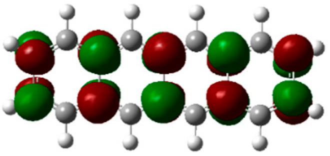	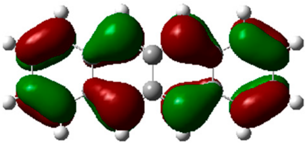	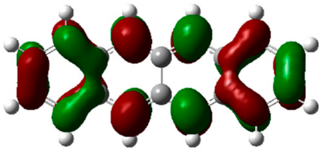
40	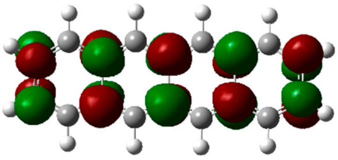	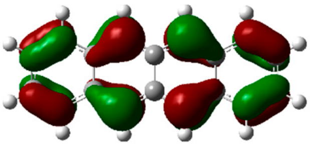	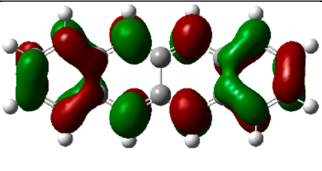

Table S9. Calculated lowest energy transitions for twisted tetracenes and their orbital contribution. The brightest state has been considered for ^1B_b transitions.

Twist angle (°)	State number	Oscillator Strength (f)	Transition energy (eV)	Major transitions			
				Orbitals involved	Contri bution (%)	Orbitals involved	Contri bution (%)
30	**1**	**0.0923**	**2.9457**	**HO→LU**	**99.20**		
	**5**	**2.9247**	**5.0306**	**HO→ LU+1**	53.06	HO-1→LU	46.13
40	**1**	**0.091**	**2.9357**	**HO→LU**	**99.17**		
	**5**	**0.0254**	**4.9999**	**HO→LU+1**	**52.93**	HO-1→LU	46.24

Table S10. Crystal data and structure refinement for **2** and **8**

**Compound**	**2**	**8**
**Empirical formula**	C40 H36 Cl4 O4	C39 H29 Cl3 O4 S4[1]
**Formula weight**	722.49	796.21
**Temperature (K)**	123(1)	123.5(1)
**Radiation**	0.71073 Å (Mo-Kα)	0.71073 Å (Mo-Kα)
**Crystal system**	Triclinic	Orthorhombic
**Space group**	P-1	Pbcn
**a (Å)**	7.548(2) Å	23.9549(8) Å
**b (Å)**	10.637(2) Å	10.5466(3) Å
**c (Å)**	11.196(3) Å	13.8395(5) Å
**α (°)**	82.224(4)°	90°
**β (°)**	85.318(4)°	90°
**γ (°)**	74.743(4)°	90°
**V (Å^3)**	858.2(3) Å3	3496.4(2) Å^3
**Z**	1	4
**D_x (mg/m^3)**	1.398	1.513
**μ (mm^{-1})**	0.387	0.544
**F(0 0 0)**	376	1640
**Crystal size (mm^3)**	0.46 × 0.13 × 0.08	0.42 × 0.12 × 0.06
**Theta range for data collection (°)**	2.00 to 25.00.	1.7 to 28
**Index ranges**	-8<=h<=8, -12<=k<=12, -13<=l<=13	-31<=h<=31, -13<=k<=13, -18<=l<=18
**Reflections collected**	5851	37674
**Independent reflections**	2910 [R(int) = 0.0266]	4177 [R(int) = 0.0966, R_{sigma} = 0.0386]
**Completeness to theta**	96.30%	97.6%

[1] A solvent mask was calculated, and 196 electrons were found in a volume of 604/Å^3 in 1 void per unit cell. This is consistent with the presence of 1[$CHCl_3$] per Formula unit, which accounts for 232 electrons per unit cell.

**Absorption correction**	MULTI-SCAN	Semi-empirical from equivalents
**Max. and min. transmission**	0.970 and 0.842	1 and 0.795
**Refinement method**	Full-matrix least-squares on F2	Full-matrix least-squares on F2
**Data / restraints / parameters**	2910 / 0 / 219	4177 / 0 / 210
**Goodness-of-fit on F2**	1.149	1.068
***R* indices I > 2σ(I)**	R1 = 0.0556, wR2 = 0.1131	R1 = 0.0687, wR2 = 0.1571
***R* indices (all data)**	R1 = 0.0625, wR2 = 0.1214	R1 = 0.0868, wR2 = 0.1671
**Largest diff. peak and hole (e Å^{-3})**	0.31 and -0.30	0.59 and -0.32
**CCDC deposition number**	2251167	2191791

S5 Analysis of C–H···π interaction

The C–H···π interaction involving H12a, C15, and C16 and the centroid of the rind (X) was understood and analyzed for compound **3**.[2]

Distance between H12a and X = 2.66Å < 3.0Å; interplanar angle (ω) for □C15-C16-H12A - □C15-C16-X = 69.69° < 127.5° and angle(θ) ∠H12a-C12- C15 = 9.47° <70°.

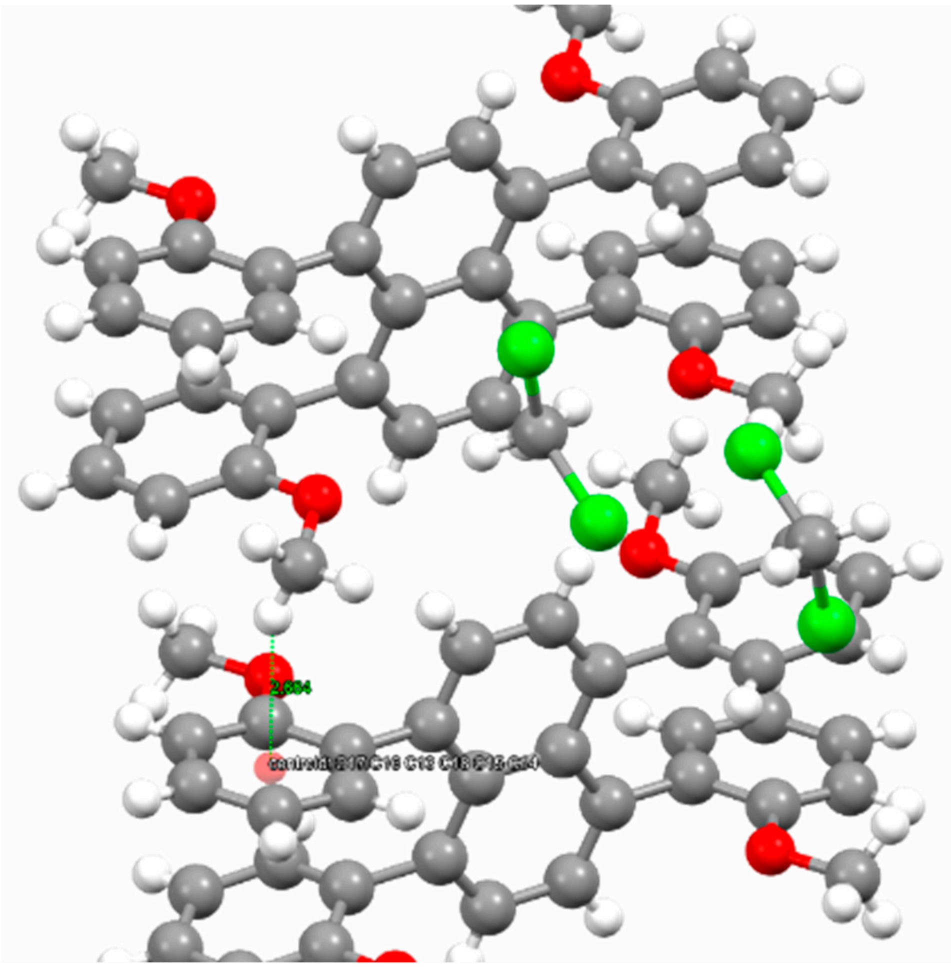

(a)

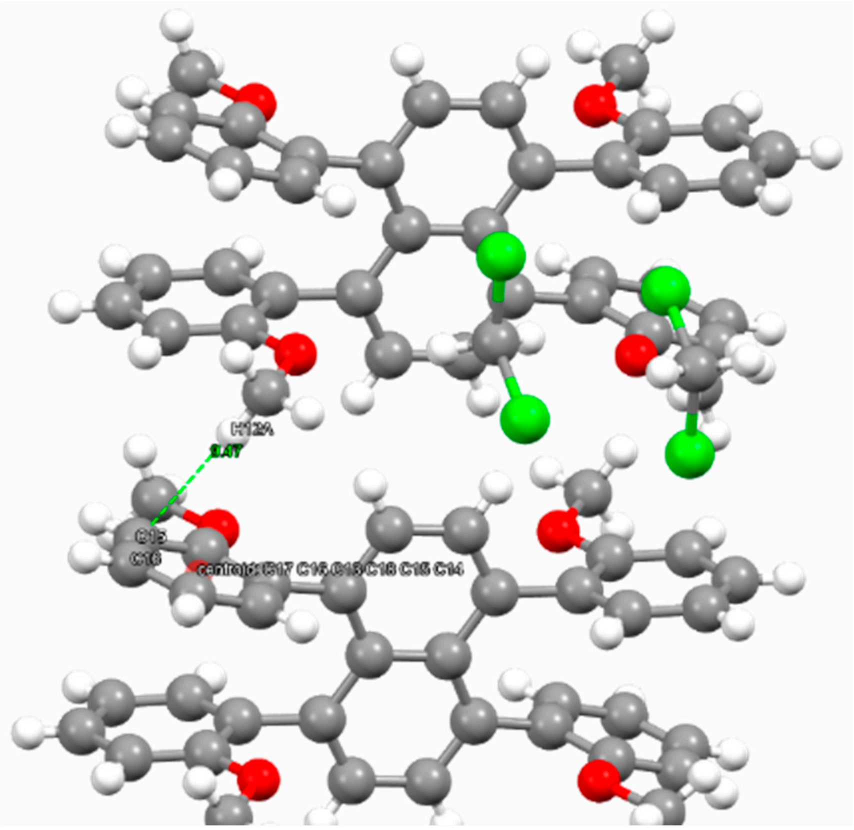

(b)

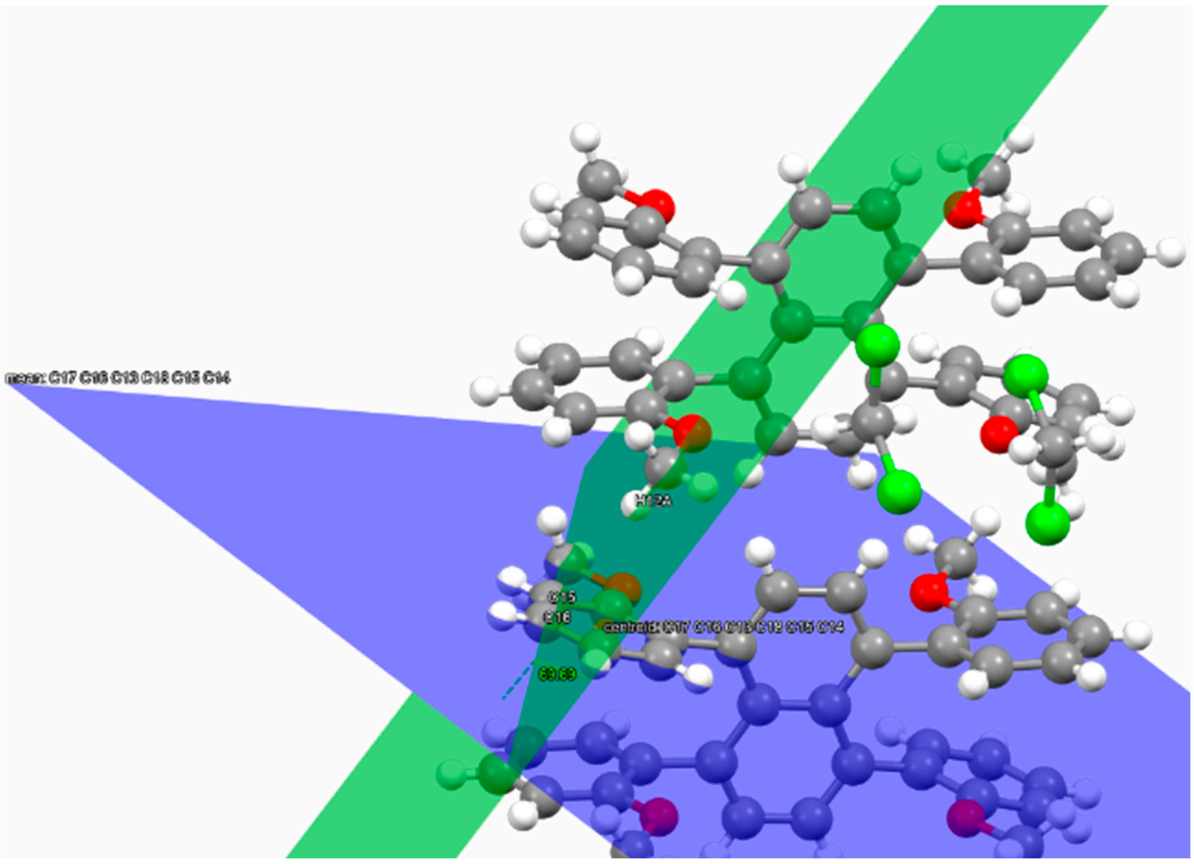

(c)

Figure S9. Important parameters for C–H···π interaction in compound **3**. (a) distance between H12 and π-centroid, (b) dihedral angle (ω) between □C15-C16-H12A - □C15-C16-X and (c) ∠H12a-C12- C15 (θ).

Table S11. ORTEP diagram for the crystals drawn at 50% probability level.

compound	Front view	View through short axis
**3**	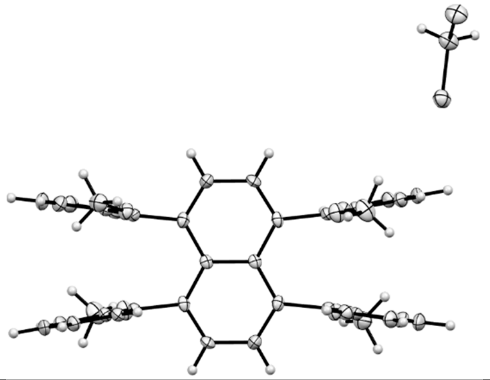	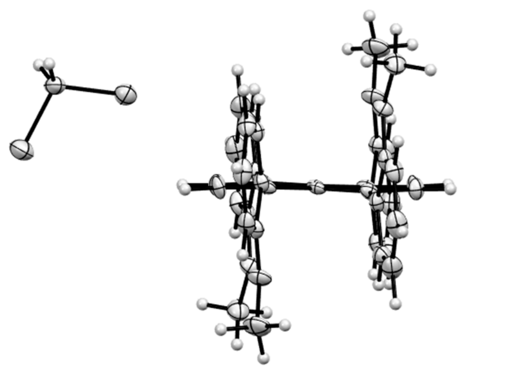
**8**	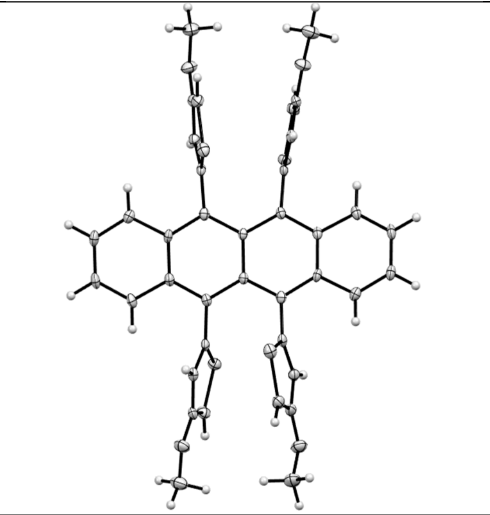	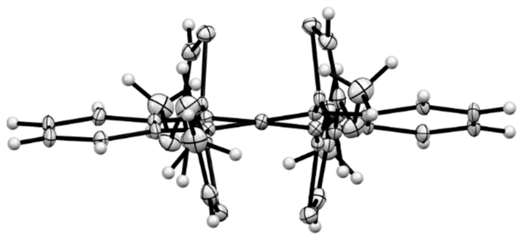

S6 Optimized coordinates of the tetraaryl-substituted tetracenes (DFT-CAMB3LYP-SDD)

M-4FTc

```
                         Standard orientation:
 ---------------------------------------------------------------------
 Center     Atomic      Atomic             Coordinates (Angstroms)
 Number     Number       Type             X           Y           Z
 ---------------------------------------------------------------------
      1          6           0        1.409934    1.239517    0.191471
      2          6           0        0.708109    2.455550    0.149051
      3          6           0        1.358457    3.726675    0.369567
      4          1           0        2.399708    3.731788    0.665108
```

5	6	0	0.688448	4.908076	0.204360
6	1	0	1.196699	5.852705	0.372587
7	6	0	-0.688462	4.908076	0.204319
8	1	0	-1.196715	5.852706	0.372538
9	6	0	-1.358468	3.726675	0.369535
10	1	0	-2.399719	3.731787	0.665075
11	6	0	-0.708116	2.455549	0.149031
12	6	0	-1.409938	1.239515	0.191461
13	6	0	-0.723495	-0.000001	0.000003
14	6	0	2.840654	1.260386	0.553847
15	6	0	3.975335	1.787388	0.005265
16	1	0	4.036959	2.247771	0.978742
17	6	0	5.052981	1.582074	0.930375
18	6	0	4.512636	0.948022	2.016714
19	1	0	4.913144	0.598517	2.953579
20	6	0	-2.840662	1.260387	0.553824
21	6	0	-3.975346	1.787343	0.005325
22	1	0	-4.036971	2.247673	0.978828
23	6	0	-5.052983	1.582120	0.930345
24	6	0	-4.512626	0.948179	2.016744
25	1	0	-4.913120	0.598790	2.953658
26	6	0	-1.409934	-1.239519	0.191457
27	6	0	-0.708109	-2.455551	0.149030
28	6	0	-1.358456	-3.726678	0.369538
29	1	0	-2.399707	-3.731793	0.665078
30	6	0	-0.688446	-4.908078	0.204326
31	1	0	-1.196696	-5.852708	0.372548
32	6	0	0.688464	-4.908074	0.204353
33	1	0	1.196718	-5.852703	0.372577
34	6	0	1.358469	-3.726671	0.369563
35	1	0	2.399720	-3.731782	0.665105
36	6	0	0.708117	-2.455548	0.149051
37	6	0	1.409938	-1.239513	0.191475
38	6	0	0.723493	0.000001	0.000003
39	6	0	-2.840658	-1.260394	0.553821
40	6	0	-3.975340	-1.787357	0.005323
41	1	0	-4.036965	-2.247692	0.978824
42	6	0	-5.052977	-1.582133	0.930347
43	6	0	-4.512621	-0.948183	2.016741
44	1	0	-4.913115	-0.598790	2.953654
45	6	0	2.840658	-1.260379	-0.553849
46	6	0	3.975340	-1.787373	0.005267
47	1	0	4.036966	-2.247749	0.978748
48	6	0	5.052987	-1.582062	-0.930373
49	6	0	4.512641	-0.948019	-2.016717
50	1	0	4.913149	-0.598520	-2.953584
51	1	0	6.086567	-1.863196	-0.800797
52	1	0	6.086560	1.863212	0.800802
53	1	0	-6.086555	1.863283	-0.800773
54	1	0	-6.086548	-1.863300	0.800777
55	8	0	-3.156545	-0.749270	1.818553
56	8	0	-3.156549	0.749268	-1.818557
57	8	0	3.156530	0.749244	1.818575
58	8	0	3.156534	-0.749243	-1.818579

***M*-4ThTc**

Standard orientation:

Center   Atomic   Atomic   Coordinates (Angstroms)

Number	Number	Type	X	Y	Z
1	6	0	1.410678	1.240247	0.219525
2	6	0	0.703674	2.455408	0.166870
3	6	0	1.346390	3.725507	0.412022
4	1	0	2.378557	3.729775	0.737530
5	6	0	0.681390	4.907115	0.226135
6	1	0	1.184102	5.851806	0.410111
7	6	0	-0.681440	4.907095	-0.226347
8	1	0	-1.184172	5.851770	-0.410347
9	6	0	-1.346417	3.725469	-0.412194
10	1	0	-2.378588	3.729705	-0.737693
11	6	0	-0.703673	2.455390	-0.167008
12	6	0	-1.410661	1.240218	-0.219600
13	6	0	-0.725168	-0.000006	-0.000015
14	6	0	2.830507	1.274681	0.640750
15	6	0	3.939721	1.767156	0.007857
16	1	0	3.910073	2.131514	-1.011431
17	6	0	5.140204	1.723076	0.802253
18	6	0	4.938750	1.215022	2.056910
19	1	0	5.657796	1.071752	2.849177
20	6	0	-2.830508	1.274626	-0.640764
21	6	0	-3.939681	1.767189	-0.007870
22	1	0	-3.909974	2.131664	1.011373
23	6	0	-5.140237	1.722892	-0.802145
24	6	0	-4.938886	1.214541	-2.056698
25	1	0	-5.658016	1.070991	-2.848838
26	6	0	-1.410643	-1.240238	0.219583
27	6	0	-0.703636	-2.455399	0.167008
28	6	0	-1.346360	-3.725485	0.412212
29	1	0	-2.378530	-3.729732	0.737711
30	6	0	-0.681364	-4.907103	0.226381
31	1	0	-1.184082	-5.851784	0.410395
32	6	0	0.681466	-4.907108	-0.226101
33	1	0	1.184193	-5.851794	-0.410064
34	6	0	1.346448	-3.725493	-0.412004
35	1	0	2.378614	-3.729749	-0.737513
36	6	0	0.703712	-2.455400	-0.166870
37	6	0	1.410697	-1.240228	-0.219542
38	6	0	0.725204	0.000005	-0.000014
39	6	0	-2.830487	-1.274662	0.640756
40	6	0	-3.939653	-1.767274	0.007887
41	1	0	-3.909942	-2.131791	-1.011341
42	6	0	-5.140204	-1.722974	0.802167
43	6	0	-4.938859	-1.214568	2.056699
44	1	0	-5.657987	-1.071004	2.848837
45	6	0	2.830529	-1.274647	-0.640758
46	6	0	3.939749	-1.767077	-0.007842
47	1	0	3.910103	-2.131399	1.011458
48	6	0	5.140235	-1.722998	-0.802233
49	6	0	4.938777	-1.214993	-2.056909
50	1	0	5.657825	-1.071734	-2.849177
51	1	0	6.106319	-2.053961	-0.441028
52	1	0	6.106282	2.054075	0.441066
53	1	0	-6.106314	2.053836	-0.440904
54	1	0	-6.106275	-2.053956	0.440945
55	16	0	-3.242770	-0.765167	2.316710
56	16	0	-3.242788	0.765195	-2.316739
57	16	0	3.242723	0.765291	2.316755
58	16	0	3.242741	-0.765311	-2.316781

***M*-4SeTc**

Standard orientation:

Center Number	Atomic Number	Atomic Type	Coordinates (Angstroms) X	Y	Z
1	6	0	1.410212	1.238641	0.236054
2	6	0	0.701270	2.454045	0.178615
3	6	0	1.338184	3.723752	0.438761
4	1	0	2.363535	3.727632	0.785566
5	6	0	0.676891	4.905818	0.240340
6	1	0	1.175963	5.850205	0.435331
7	6	0	-0.675824	4.905925	-0.240839
8	1	0	-1.174696	5.850389	-0.435971
9	6	0	-1.337370	3.723971	-0.439072
10	1	0	-2.362727	3.728011	-0.785862
11	6	0	-0.700725	2.454169	-0.178729
12	6	0	-1.409944	1.238920	-0.235960
13	6	0	-0.724890	0.000056	0.000117
14	6	0	2.819413	1.265421	0.694562
15	6	0	3.936592	1.768345	0.086472
16	1	0	3.910121	2.144192	-0.930019
17	6	0	5.149655	1.733207	0.866406
18	6	0	5.011452	1.222359	2.127849
19	1	0	5.782193	1.103416	2.877136
20	6	0	-2.819114	1.265959	-0.694554
21	6	0	-3.936158	1.769554	-0.086771
22	1	0	-3.909611	2.145912	0.929528
23	6	0	-5.149187	1.734455	-0.866758
24	6	0	-5.011088	1.222944	-2.127944
25	1	0	-5.781825	1.103910	-2.877221
26	6	0	-1.410208	-1.238685	0.236067
27	6	0	-0.701257	-2.454084	0.178656
28	6	0	-1.338173	-3.723788	0.438813
29	1	0	-2.363527	-3.727661	0.785611
30	6	0	-0.676881	-4.905856	0.240403
31	1	0	-1.175954	-5.850241	0.435400
32	6	0	0.675833	-4.905968	-0.240779
33	1	0	1.174703	-5.850434	-0.435904
34	6	0	1.337379	-3.724016	-0.439028
35	1	0	2.362733	-3.728062	-0.785825
36	6	0	0.700736	-2.454211	-0.178697
37	6	0	1.409948	-1.238958	-0.235947
38	6	0	0.724889	-0.000099	0.000123
39	6	0	-2.819408	-1.265480	0.694577
40	6	0	-3.936563	-1.768532	0.086547
41	1	0	-3.910073	-2.144500	-0.929899
42	6	0	-5.149623	-1.733377	0.866483
43	6	0	-5.011441	-1.222393	2.127873
44	1	0	-5.782182	-1.103423	2.877156
45	6	0	2.819117	-1.265980	-0.694549
46	6	0	3.936173	-1.769558	-0.086774
47	1	0	3.909636	-2.145927	0.929521
48	6	0	5.149199	-1.734428	-0.866764
49	6	0	5.011090	-1.222892	-2.127939
50	1	0	5.781825	-1.103824	-2.877212
51	1	0	6.099117	-2.084223	-0.477436
52	1	0	6.099665	2.082484	0.476841

53	1	0	-6.099097	2.084265	-0.477424
54	1	0	-6.099614	-2.082759	0.476965
55	34	0	-3.228455	-0.701259	2.481186
56	34	0	3.228430	0.701406	2.481244
57	34	0	3.228228	-0.701133	-2.480944
58	34	0	-3.228240	0.701139	-2.480955

***M*-4TeTc**

Standard orientation:

Center Number	Atomic Number	Atomic Type	Coordinates (Angstroms) X	Y	Z
1	6	0	1.410556	0.248370	-1.237390
2	6	0	0.699375	0.185073	-2.452821
3	6	0	1.330922	0.456663	-3.722604
4	1	0	2.350872	0.818669	-3.726842
5	6	0	0.672416	0.248724	-4.905021
6	1	0	1.168352	0.451965	-5.849377
7	6	0	-0.672131	-0.253870	-4.904802
8	1	0	-1.167995	-0.458119	-5.848979
9	6	0	-1.330719	-0.460545	-3.722209
10	1	0	-2.350670	-0.822552	-3.726130
11	6	0	-0.699263	-0.187603	-2.452665
12	6	0	-1.410534	-0.249626	-1.237210
13	6	0	-0.724957	0.000018	-0.000033
14	6	0	2.810312	0.740604	-1.259753
15	6	0	3.923502	0.134503	-1.773327
16	1	0	3.877456	-0.885021	-2.144334
17	6	0	5.169496	0.870360	-1.777892
18	6	0	5.149177	2.150080	-1.294309
19	1	0	6.006549	2.808805	-1.237986
20	6	0	-2.810275	-0.741936	-1.259144
21	6	0	-3.923479	-0.136377	-1.773331
22	1	0	-3.877456	0.882770	-2.145376
23	6	0	-5.169442	-0.872283	-1.777200
24	6	0	-5.149086	-2.151521	-1.292339
25	1	0	-6.006435	-2.810223	-1.235391
26	6	0	-1.410588	0.249639	1.237111
27	6	0	-0.699392	0.187592	2.452596
28	6	0	-1.330937	0.460490	3.722098
29	1	0	-2.350890	0.822493	3.725963
30	6	0	-0.672428	0.253773	4.904728
31	1	0	-1.168363	0.457983	5.848875
32	6	0	0.672120	-0.248817	4.905023
33	1	0	1.167986	-0.452095	5.849409
34	6	0	1.330707	-0.456710	3.722642
35	1	0	2.350656	-0.818716	3.726936
36	6	0	0.699247	-0.185077	2.452817
37	6	0	1.410502	-0.248354	1.237416
38	6	0	0.724910	0.000015	-0.000004
39	6	0	-2.810332	0.741927	1.259019
40	6	0	-3.923510	0.136385	1.773276
41	1	0	-3.877472	-0.882758	2.145332
42	6	0	-5.169486	0.872277	1.777144
43	6	0	-5.149156	2.151500	1.292248
44	1	0	-6.006514	2.810190	1.235293
45	6	0	2.810258	-0.740599	1.259807
46	6	0	3.923482	-0.134433	1.773234

47	1	0	3.877456	0.885129	2.144143
48	6	0	5.169468	-0.870295	1.777830
49	6	0	5.149117	-2.150072	1.294392
50	1	0	6.006483	-2.808809	1.238112
51	1	0	6.082508	-0.413256	2.148664
52	1	0	6.082513	0.413358	-2.148830
53	1	0	-6.082468	-0.415685	-2.148609
54	1	0	-6.082497	0.415680	2.148595
55	52	0	-3.263867	-2.698475	-0.658766
56	52	0	-3.263932	2.698503	0.658707
57	52	0	3.263941	2.697773	-0.661399
58	52	0	3.263874	-2.697782	0.661545

***M*-8**

Standard orientation:

Center Number	Atomic Number	Atomic Type	Coordinates (Angstroms) X	Y	Z
1	6	0	1.403057	-1.198182	-0.258415
2	6	0	0.699105	-2.412595	-0.180824
3	6	0	1.340616	-3.682699	-0.430861
4	1	0	2.368193	-3.686151	-0.771130
5	6	0	0.678961	-4.864261	-0.234364
6	1	0	1.179400	-5.809087	-0.423776
7	6	0	-0.678881	-4.864303	0.234033
8	1	0	-1.179279	-5.809161	0.423391
9	6	0	-1.340576	-3.682779	0.430626
10	1	0	-2.368140	-3.686294	0.770929
11	6	0	-0.699118	-2.412634	0.180670
12	6	0	-1.403060	-1.198223	0.258426
13	6	0	-0.726494	0.047285	0.032402
14	6	0	2.820565	-1.251400	-0.687097
15	6	0	3.929218	-1.658202	0.001254
16	1	0	3.886401	-1.935301	1.045559
17	6	0	5.140236	-1.620894	-0.783142
18	6	0	4.943742	-1.229228	-2.078446
19	6	0	-2.820521	-1.251393	0.687268
20	6	0	-3.929263	-1.658234	-0.000930
21	1	0	-3.886560	-1.935360	-1.045234
22	6	0	-5.140175	-1.620912	0.783611
23	6	0	-4.943531	-1.229145	2.078870
24	1	0	-5.686640	-1.113600	2.850810
25	6	0	-1.420481	1.292808	-0.131483
26	6	0	-0.713281	2.507683	-0.115110
27	6	0	-1.376258	3.777777	-0.299712
28	1	0	-2.432764	3.782720	-0.534156
29	6	0	-0.698696	4.958930	-0.166046
30	1	0	-1.216238	5.903651	-0.301912
31	6	0	0.698437	4.958945	0.166499
32	1	0	1.215945	5.903676	0.302427
33	6	0	1.376046	3.777806	0.300058
34	1	0	2.432560	3.782767	0.534466
35	6	0	0.713110	2.507701	0.115375
36	6	0	1.420396	1.292871	0.131575
37	6	0	0.726486	0.047313	-0.032345
38	6	0	-2.869761	1.344549	-0.436186
39	6	0	-3.919632	1.754675	0.330311
40	1	0	-3.834678	2.048661	1.367377
41	6	0	-5.189144	1.704840	-0.348487

42	6	0	-5.107214	1.292797	-1.653201
43	1	0	-5.890957	1.179021	-2.384713
44	6	0	2.869714	1.344740	0.436085
45	6	0	3.919475	1.754705	-0.330639
46	1	0	3.834386	2.048495	-1.367750
47	6	0	5.189094	1.704978	0.347985
48	6	0	5.107336	1.293250	1.652804
49	1	0	5.891163	1.179652	2.384254
50	16	0	-3.416362	0.950107	-2.107340
51	16	0	-3.232687	-0.872401	2.392149
52	16	0	3.232900	-0.872677	-2.391999
53	16	0	3.416557	0.950583	2.107234
54	8	0	6.317793	2.053795	-0.361902
55	8	0	-6.413244	-1.909578	0.309090
56	8	0	-6.317938	2.053903	0.361109
57	8	0	6.413267	-1.909474	-0.308431
58	1	0	5.686942	-1.113715	-2.850304
59	6	0	6.511330	-2.516208	1.002537
60	1	0	6.149229	-1.835020	1.782623
61	1	0	7.572425	-2.717515	1.155657
62	1	0	5.950018	-3.458210	1.047227
63	6	0	7.595931	1.771747	0.255660
64	1	0	7.722669	2.349973	1.180622
65	1	0	8.351050	2.072923	-0.470706
66	1	0	7.686853	0.698568	0.465752
67	6	0	-6.511540	-2.516260	-1.001887
68	1	0	-6.149250	-1.835170	-1.781968
69	1	0	-5.950512	-3.458438	-1.046619
70	1	0	-7.572697	-2.717261	-1.155006
71	6	0	-7.595973	1.771979	-0.256650
72	1	0	-7.722234	2.349667	-1.182019
73	1	0	-7.687212	0.698695	-0.466051
74	1	0	-8.351188	2.073902	0.469307

***M*-rubrene**

Standard orientation:

Center Number	Atomic Number	Atomic Type	Coordinates (Angstroms) X	Y	Z
1	6	0	-1.413204	-1.239824	0.216602
2	6	0	-0.706180	-2.452466	0.159508
3	6	0	-1.353979	-3.725805	0.383971
4	1	0	-2.392684	-3.735123	0.688344
5	6	0	-0.688334	-4.907889	0.204817
6	1	0	-1.197892	-5.852539	0.370016
7	6	0	0.688697	-4.908090	-0.203832
8	1	0	1.198188	-5.852827	-0.368679
9	6	0	1.354336	-3.725996	-0.383317
10	1	0	2.393061	-3.735329	-0.687641
11	6	0	0.706511	-2.452527	-0.159304
12	6	0	1.413408	-1.239800	-0.216805
13	6	0	0.726234	-0.000003	-0.000123
14	6	0	1.413302	1.239774	0.216731
15	6	0	0.706253	2.452472	0.159546
16	6	0	1.353880	3.725916	0.384185
17	1	0	2.392565	3.735269	0.688646
18	6	0	0.688115	4.908016	0.205117
19	1	0	1.197483	5.852739	0.370414
20	6	0	-0.688861	4.907816	-0.203668

21	1	0	-1.198547	5.852464	-0.368479
22	6	0	-1.354335	3.725705	-0.383359
23	1	0	-2.393022	3.735008	-0.687790
24	6	0	-0.706389	2.452370	-0.159359
25	6	0	-1.413299	1.239654	-0.216965
26	6	0	-0.726150	-0.000057	-0.000227
27	6	0	-2.837119	-1.271765	0.686172
28	6	0	-3.885775	-1.789236	-0.090721
29	6	0	-3.111815	-0.862692	2.003389
30	6	0	-5.183557	-1.875886	0.426174
31	1	0	-3.685975	-2.108600	-1.109212
32	6	0	-4.407124	-0.955859	2.528516
33	1	0	-2.303438	-0.476858	2.618079
34	6	0	-5.450005	-1.461134	1.739588
35	1	0	-5.984956	-2.265380	-0.194882
36	1	0	-4.600076	-0.639765	3.549873
37	1	0	-6.456290	-1.532875	2.142434
38	6	0	-2.837173	1.271541	-0.686657
39	6	0	-3.111841	0.863233	-2.004059
40	6	0	-3.885941	1.788296	0.090655
41	6	0	-4.407217	0.956520	-2.529077
42	1	0	-2.303447	0.477928	-2.619062
43	6	0	-5.183742	1.875022	-0.426099
44	1	0	-3.686112	2.107019	1.109342
45	6	0	-5.450150	1.461080	-1.739807
46	1	0	-4.600147	0.641037	-3.550627
47	1	0	-5.985202	2.263935	0.195241
48	1	0	-6.456457	1.532874	-2.142589
49	6	0	2.837251	-1.271492	-0.686601
50	6	0	3.886164	-1.788670	0.090161
51	6	0	3.111663	-0.862365	-2.003862
52	6	0	5.183889	-1.875030	-0.426913
53	1	0	3.686583	-2.107940	1.108725
54	6	0	4.406916	-0.955233	-2.529162
55	1	0	2.303108	-0.476676	-2.618409
56	6	0	5.450034	-1.460255	-1.740380
57	1	0	5.985485	-2.264297	0.194032
58	1	0	4.599670	-0.639090	-3.550541
59	1	0	6.456265	-1.531794	-2.143398
60	6	0	2.837135	1.271618	0.686505
61	6	0	3.111737	0.862673	2.003737
62	6	0	3.885949	1.788728	-0.090500
63	6	0	4.407083	0.955708	2.528877
64	1	0	2.303325	0.477018	2.618495
65	6	0	5.183715	1.875206	0.426374
66	1	0	3.686197	2.107853	-1.109077
67	6	0	5.450054	1.460650	1.739907
68	1	0	4.599949	0.639710	3.550281
69	1	0	5.985219	2.264399	-0.194734
70	1	0	6.456336	1.532299	2.142775

S7 Optimized coordinates of the twisted parent tetracenes (DFT-CAMB3LYP-SDD)

M-Tc-30

Standard orientation:

Center	Atomic	Atomic	Coordinates (Angstroms)		

Number	Number	Type	X	Y	Z
1	6	0	-3.717126	1.385927	0.279456
2	1	0	-3.714523	2.449495	0.503269
3	6	0	-2.450894	0.719125	0.093661
4	6	0	-2.450328	-0.718418	-0.093668
5	6	0	-3.715474	-1.386540	-0.279558
6	1	0	-3.711863	-2.450071	-0.503432
7	6	0	-4.895630	-0.697038	-0.186692
8	1	0	-5.843108	-1.205891	-0.336814
9	6	0	-4.896961	0.695282	0.186601
10	1	0	-5.845056	1.202954	0.336579
11	6	0	-1.237160	-1.405642	-0.094569
12	1	0	-1.236565	-2.490188	-0.174753
13	6	0	0.000001	-0.723722	0.000009
14	6	0	0.000001	0.725375	-0.000009
15	6	0	-1.237720	1.406859	0.094567
16	1	0	-1.237544	2.491417	0.174749
17	6	0	1.237716	1.406858	-0.094566
18	1	0	1.237551	2.491416	-0.174742
19	6	0	1.237156	-1.405641	0.094569
20	1	0	1.236570	-2.490188	0.174748
21	6	0	2.450330	-0.718415	0.093674
22	6	0	2.450896	0.719122	-0.093667
23	6	0	3.715472	-1.386540	0.279554
24	1	0	3.711866	-2.450071	0.503427
25	6	0	4.895631	-0.697035	0.186693
26	6	0	4.896962	0.695279	-0.186602
27	6	0	3.717124	1.385926	-0.279452
28	1	0	3.714526	2.449494	-0.503264
29	1	0	5.843106	-1.205891	0.336818
30	1	0	5.845054	1.202954	-0.336583

***M*-Tc-40**

Standard orientation:

Center Number	Atomic Number	Atomic Type	Coordinates (Angstroms) X	Y	Z
1	6	0	-3.714286	1.363792	0.370640
2	1	0	-3.711516	2.409439	0.667108
3	6	0	-2.448730	0.714730	0.124587
4	6	0	-2.447698	-0.713409	-0.124636
5	6	0	-3.711280	-1.364901	-0.370875
6	1	0	-3.706660	-2.410490	-0.667397
7	6	0	-4.890862	-0.678940	-0.246897
8	1	0	-5.837618	-1.172524	-0.445003
9	6	0	-4.893248	0.675674	0.246658
10	1	0	-5.841148	1.167004	0.444525
11	6	0	-1.235773	-1.402779	-0.125795
12	1	0	-1.234672	-2.485011	-0.232505
13	6	0	0.000005	-0.723052	0.000006
14	6	0	0.000005	0.726082	-0.000006
15	6	0	-1.236792	1.405033	0.125779
16	1	0	-1.236438	2.487285	0.232492
17	6	0	1.236783	1.405032	-0.125787
18	1	0	1.236444	2.487284	-0.232499
19	6	0	1.235764	-1.402777	0.125802
20	1	0	1.234677	-2.485009	0.232512
21	6	0	2.447704	-0.713404	0.124640

22	6	0	2.448737	0.714724	-0.124591
23	6	0	3.711274	-1.364899	0.370871
24	1	0	3.706663	-2.410491	0.667382
25	6	0	4.890864	-0.678936	0.246891
26	6	0	4.893250	0.675670	-0.246651
27	6	0	3.714280	1.363790	-0.370635
28	1	0	3.711520	2.409440	-0.667093
29	1	0	5.837615	-1.172534	0.444987
30	1	0	5.841145	1.167014	-0.444509

S8 Reference

[1] Bedi, A.; Gidron, O. Chiroptical Properties of Twisted Acenes: Experimental and Computational Study. *Chem. Eur. J.* **2019**, *25*, 3279-3285.

[2] M. Nishio, Y. Umezawa, M. Hirota, Y. Takeuchi, The CH/π interaction: Significance in molecular recognition, Tetrahedron 51 (1995) 8665-8671.
